# Soil bacterial communities of Sahara and Gibson deserts: Physiological and taxonomical characteristics

**DOI:** 10.3934/microbiol.2018.4.685

**Published:** 2018-12-17

**Authors:** Andrey A. Belov, Vladimir S. Cheptsov, Elena A. Vorobyova

**Affiliations:** 1 Soil Science Faculty, Lomonosov Moscow State University, Moscow, Russia; 2 Space Research Institute, Russian Academy of Sciences, Moscow, Russia

**Keywords:** desert, extremotolerance, stress-tolerance, culturable bacterial community, antibiotic resistance, soil

## Abstract

The purpose of this research was to investigate the structure of soil bacteria communities present in the Gibson (Australia) and the Sahara (Egypt) deserts, as well as to estimate strain survivability under different environmental factors. It should be noticed that the screening of bacterial resistance to wide spectra of principally different stress conditions was performed for the first time. Experiments were conducted with culturable bacterial communities. Strains were identified using 16S rRNA sequencing, and stress-tolerance was estimated by growing strains in various nutrient media. In order to characterize the community the epifluorescent microscopy and multisubstrate testing were also performed. High bacterial abundance in the desert soils was detected, and there was seen a significant proportion of culturable cells. The close numbers of psychotropic and mesophilic bacteria in arid ecosystems were revealed. The representatives of the *Actinobacteria* phylum were dominant in the microbial communities, and *Firmicutes*, *Proteobacteria*, and *Bacteroidetes* phyla representatives were also identified. Tolerance of the axenic bacterial cultures, isolated from arid desert ecotopes, to temperature, pH, salts (KCl, NaCl, MgSO_4_, NaHCO_3_), strong oxidizers (Mg(ClO_4_)_2_), and antibiotics (ampicillin, cephalexin, chloramphenicol, tetracycline, doxycycline, kanamycin, rifampicin) was studied. The bacterial isolates were characterized by polyextremotolerance and by the ability to maintain metabolic activity in vitro while influenced by a wide range of physicochemical and biotic factors.

## 1. Introduction

A significant part of the land surface of our planet is covered with hot deserts and semi-deserts [1]. The high rates of aridization and land degradation are shown, including those occurring as a result of high intensity of industrial and agricultural use [2]. Due to the large areas, such ecosystems play a significant role in the biogeochemical cycles of various chemical elements and affect gas composition of the atmosphere [3]. Also, deserts are the habitats of organisms, that have adapted their metabolism to extremely heterogeneous and changing environmental conditions [4]. Such organisms are often valuable to biotechnology, for example, as the producers of industrial substances [5],[6]. Considering the large area of arid habitats and its insufficient study, it is likely that the larger part of unexplored microorganisms' biodiversity is still concealed in such ecotopes [6].

The studies of arid ecotopes are also a source of knowledge of the microorganism's stability under extreme conditions and of the microbes' stress adaptation mechanisms [5],[7]. This data is currently used in astrobiological studies. A number of terrestrial deserts are considered as analogues of extraterrestrial objects due to the similarity of certain physicochemical factors, such as strong drying, high insolation, presence of salts and strong oxidants, and the similarity of mineralogical composition. In particular, the Atacama (Chile), Negev (Israel), Mojave (USA) Deserts, and others are such analog objects for astrobiological research [8].

Investigation of microbial communities in desert ecosystems is necessary to reveal desertification mechanisms and reduce aridization rates [3]. Microorganisms play a leading role in primary successions and have a significant effect on primary soil formation, but dating their role in primary soil formation and their impact on further soil cover genesis has been insufficiently studied [9]–[11].

Deserts differ considerably in physicochemical conditions, but they represent extreme habitats for microorganisms due to contrast changes in temperature, humidity, and pH, low abundance of nutrients, high salt concentrations, extreme solar and wind impacts [12],[13]. Because of the deserts' high diversity and vast areas, the microbiological studies were conducted only for a limited number of deserts ecotopes [14],[15]. To date, the studies of microbial communities of arid ecosystems were locally performed with the use of predominantly molecular-genetic investigational methods.

The Atacama Desert is one of the most investigated arid ecosystems. Surface and subsurface soil samples from the most arid region within the Atacama Desert (Yungay, Chile) showed the presence of 2–10 × 10^6^ bacteria cell equivalents per gram of soil (obtained by phospholipids fatty acids (PLFA) analysis). Cultured bacteria number varied from 10^2^ to 10^4^ colony forming units (CFU) per gram [16]. Cultivated bacteria from the Atacama Desert belonged to *Actinobacteria*, *Firmicutes* and *Proteobacteria* phyla [16]. By the denaturing gradient gel electrophoresis (DGGE) analysis high bacterial diversity dominated with *Actinobacteria* (*Actinobacterium*, *Aciditerrimonas* and *Geodermatophilus*), *Proteobacteria* (*Caulobacter*, *Sphingomonas*), Firmicutes (*Firmicutes*, *Clostridiales*) and *Acidobacteria* (*Acidobacterium*) was registered [17].

Soil studies in north-eastern Sahara (Egypt) found 10^8^ copies/g of 16S rRNA gene with quantitive PCR (q-PCR) method and about 10^4^ CFU/g of cultured aerobic bacteria [13]. In cultured bacterial community *Proteobacteria* (46.0%, predominantly *Ochrobactrum*), *Actinobacteria* (20.7%, predominantly *Rhodococcus*), and *Firmicutes* (11.3%, predominantly *Bacillus*) were the dominant phyla; *Acidobacteria* (7.9%), *Bacteroidetes* (4.6%), *Gemmatimonadetes* (1.4%), *Planctomycetes* (1.1%), and *Deinococcus-Thermus* (1.1%) were the minor phyla.

The transportation of desert sand by wind around wide desert areas is a well-known process. In particular, the deposition of the Sahara dust particles is a frequent phenomenon in Europe (for example, in period February–March 2014). A large number of 16S rRNA gene copies (10^5^ copies/mL of meltwater) was detected in European Alps snow layers containing Saharan dust. Studies of the dust-associated bacteria revealed such representatives as *Proteobacteria*, *Actinobacteria*, and *Firmicutes* [18]. *Gemmatimonadetes* and *Deinococcus-Thermus* were also found as minor parts of the microbial community. These bacteria were suggested to be specific bioindicators of Sahara dust because of their adaptations to arid oligotrophic environments and UV radiation.

Several novel bacterial species of different phyla were isolated from the Sahara Desert samples. In particular, *Geodermatophilus siccatus* was revealed during the environmental screening of arid desert soil near Ourba, Chad. The isolate was able to grow better at temperatures ranged between 20–37 °C and pH 6.0–8.5 levels. Moreover, it showed no NaCl tolerance [19]. Two gamma- and ultraviolet (UV)-radiation tolerant strains of *Deinococcus deserti* were isolated from the mixture of sand samples collected in Morocco and Tunisia after the sand exposure to 15 kGy gamma radiation [1]. The strains did not grow in rich medium. The growth was observed at temperatures between 23 and 37 °C and at pH 6–9 with an optimum of about 7.5. The strains tolerated up to 0.5% NaCl. The resistance of the strains to spectinomycin and nalidixic acid as well as tolerance to high doses of gamma- and UV-radiation were detected. Novel actinobacterial species *Saccharothrix tamanrassetensis*, isolated from Saharan soil sample of Algeria, exhibited antimicrobial activity against diverse bacteria and fungi and was able to produce two new bioactive products [20].

There is a lack of information about bacterial communities of Australian desert soils and their stress tolerance. The phylogenetic analyses of ribosomal RNA genes' sequences retrieved from desert soil of Sturt National Park (north-west Australia) indicated that the most abundant clone group was related to *Rubrobacter* [21]. In arid soil of north-east Australia, the *Thermus* species were observed as dominants [22]. It is known that these species prefer high-temperature and alkaline hot springs with low mineral salt content, and they are sensitive to NaCl, bivalent cations and sulfur compounds.

Bacterial communities of saline lake sediments and adjacent biological soil crusts (BSC) of Southern Australia as well as dust originated from these sources were studied [23]. The 16S rRNA gene sequences were affiliated with 21 phyla, among which *Cyanobacteria*, *Proteobacteria*, *Actinobacteria*, and *Firmicutes* phyla were the most abundant in all samples. The representatives of *Azospirillum*, *Bacillus*, *Clostridium*, *Defluviicoccus*, *Delftia*, *Magnetospirillum*, *Microvirga*, *Patulibacter*, *Pseudomonas*, *Rhodovibrio*, *Roseomonas*, *Rubellimicrobium*, *Rubrobacter*, *Skermanella*, *Staphylococcus*, and *Thermoleophilum* were identified. The remaining groups were less dominant and were presented by *Bacteriodetes*, *Deinococcus*, *Sphingobacteria*, *Planctomycetes*, *Chloroflexi*, *Gemmatimonadetes*, *Thermomicrobia*, *Chlorobia*, *Spartobacteria*, *Caldilineae*, *Solibacteres*, and *Ktedonobacteria*.

Microbiological analyses of the dust plume across South Australia and New South Wales found *in situ* representatives of nine phyla and 27 bacterial families with a high number of nucleotide sequences similar to uncultured organisms in the GenBank database [24]. In cultured bacterial community of dust, the *Actinobacteria* were revealed as the most common phylum (36% of isolates were related to the *Geodermatophilaceae* family). *Firmicutes* abundance *in situ* was low in contrast to the cultured data, in which *Firmicutes* were one of the most abundant groups, represented by *Bacillaceae* family. *Proteobacteria* were presented by *Oxalobacteraceae* family (mainly by *Massilia*). The majority of the cultured bacteria taxa was related to widespread soil organisms. Furthermore, pathogenic bacteria such as *Erwinia* sp., *Arthrobacter oxydans*, and *Bacillus licheniformis* were revealed, which means that desert ecosystems could contain some other pathogenic bacteria.

Despite the widely known fact that there are stressful conditions for microorganisms in deserts [25], to date, there are very few studies of bacterial tolerance of isolated strains from arid ecotopes to the broad spectrum of stress effects. High resistant to different stresses strains were isolated from desert ecosystems previously [1],[26]–[28]. Specific physiological stress-resistance mechanisms and unique metabolic processes of strains (for example, representatives of *Actinobacteria*), isolated from desert soils were shown previously [6],[17],[29]–[35].

This research focused on bacterial communities that are present in soils of the Sahara Desert (Egypt) and the central part of the Gibson Desert (Australia) which were previously poorly studied. We investigated the dependence of taxonomic structure of culturable bacterial communities on the temperature conditions of culturing, as well as the tolerance of 69 bacterial strains isolated from the above-mentioned habitats to a wide spectrum of stress factors. The results of the study contribute to the body of knowledge of desert microbial biomass, taxonomic and physiological diversity, and stress-tolerance.

## 2. Materials and methods

### 2.1. Samples description

Soil samples were collected in the Sahara Desert (South-Western Egypt, 24°25′34.6″ N 27°57′01.3″ E) and in the Gibson Desert (Western Australia 24°57′47.9″ S 125°30′28.8″ E) at the depth of 0–5 cm. Approximately 100 g of every soil was placed in sterile polypropylene tubes using a sterile scoop. The samples were stored at room temperature.

The Sahara Desert sample was represented by practically clear quartz sand, pH of aqueous extract was 7.2 ± 0.1; the Gibson Desert sample consisted of quartz sand and non-clay iron-containing minerals, pH of aqueous extract was 8.3 ± 0.1. The Sahara Desert is in general characterized by soil pH is 7.6–7.9, soil total organic carbon content is 0.1–1.2%, soil nitrogen content is 0.8–0.1% [15]. Approximate temperature range is between −5 and 45 °C and approximate precipitation is 5–150 mm/year which corresponds to hyperarid-arid climate type. The mean annual temperature of studied region is 27–28 °C [36]. The Gibson Desert is in general characterized by soil pH is 6.5–7.5, soil total organic carbon content is 0.06%, and soil nitrogen content is 0.05–0.07%. Approximate temperature range is between 6 and 40 °C and approximate precipitation is 200–400 mm/year which corresponds to semiarid climate type [15]. The mean annual temperature of studied region is 21–24 °C [37].

### 2.2. Culturing

The number of culturable heterotrophic aerobic bacteria was determined by plating sample dilutions (in sterile 0.01 M phosphate buffer solution, pH 7.45, 0.01 M KH_2_PO_4_ + K_2_HPO_4_, 0.137 M NaCl) on solid glucose-peptone-yeast (GPY) agar medium (glucose—1 g/L, peptone—2 g/L, yeast extract—1 g/L, tryptone—1 g/L, agar—20 g/L) and modified Czapek (CM) agar medium (glucose—2 g/L, sucrose—2 g/L, starch—2 g/L, NaNO_3_—2 g/L, KH_2_PO_4_—1 g/L, MgSO_4_—0.5 g/L, KCl—0.2 g/L, agar—20 g/L) in three replicates. The plate incubations were carried out at 10 °C, 25 °C, or 50 °C for ten days. Incubation time was chosen on the basis of preliminary experiments results—the process of colony formation was finished in course of ten days of incubation in described conditions. Incubation temperatures were chosen based on psychrophilic, mesophilic, and thermophilic temperature optimums [38]. Standard sterility controls were performed. Cell numbers were calculated per dry sample weight.

Isolated bacterial strains were deposited to the Astrobiological Collection of Microorganisms (Table S1) of the National Depository Bank of Live Systems “Noah's Ark” (Available from: https://depo.msu.ru).

### 2.3. Epifluorescence microscopy

The total number of prokaryotic cells in the soil samples in situ was determined by epifluorescence microscopy with acridine orange using luminescent microscope Biomed-6 PR LUM (Russia) in six replicates as described [39]

### 2.4. Multisubstrate testing

Potential metabolic activity of soil microbial communities was assessed by multisubstrate testing (MST) [40],[41] by using a set of 47 test substrates which belong to different classes of organic compounds including sugars, alcohols, amino acids, salts of organic acids, and polymers. Soil diluted suspension were added into 96-well plate, containing test substrates and triphenyltetrazolium chloride as an indicator of dehydrogenase activity in two replicates. After two days of incubation formazan concentrations in wells were measured. Complete protocol is described in [39].

### 2.5. Bacterial isolates identification

Identification of cultured heterotrophic bacteria was carried out by analysing the nucleotide sequences of the 16S rRNA gene. To conduct genomic DNA extraction, the bacterial biomass in exponential or early stationary phase of growth (two–three-day cultures) was collected from plates and suspended in the Tris-EDTA buffer (10 mM Tris, 1 mM EDTA, pH 8.0) with addition of 5% Triton X-100. Cells suspensions were vortexed at 2100 rpm for 5 min and boiled at 100 °C for 15 min. About 100 mg of sterile glass beads (250–300 µm in diameter) was added to each suspension, and the suspensions were homogenized using Minilys homogenizer (Bertin Instruments, France) at 5000 rpm for 30 s. After that lysates were centrifuged at 13400 rpm for 3 minutes and supernatant was used as DNA-matrix for PCR.

Polymerase chain reaction (PCR) was performed with primers 63f + 1387r [42], 27f + Un1492r [43],[44], 341f + 805r [45], and 27f + 537r [43],[46]. For every strain 16S rRNA gene amplification was started with 63f + 1387r primer pair. For the strains, which DNA was not amplified with 63f + 1387r primers the 27f + Un1492r primers were chosen. For the strains, whose DNA was not amplified with both 63f + 1387r and 27f + 1492r primer systems, the 341f + 805r primers were used instead. If DNA was not amplified with the three primers systems mentioned above, the 27f + 537r primer system was applied (Table S2). A PCR inhibition test was used on all DNA samples that could not be amplified by the described primer systems. Studied lysate aliquot was mixed with control *Escherichia coli* lysate (3:1), and PCR was performed. If the inhibition, where DNA concentration was lower than in positive control (simultaneous PCR with pure *E. coli* lysate) occurred, the lysate was purified using Cleanup Mini Kit (Evrogen, Russia) following the manufacturer's instructions. Purified lysates were used for further amplification.

The obtained PCR products were purified and sequenced by the Research and Production Company “Synthol” (Moscow, Russia) using the 1100r [46], 805r [45], or 537r [46] primers (Table S2). The editing of the nucleotide sequences was carried out using Chromas Lite 2.01 (http://www.technelysium.com.au). For alignment, comparison and identification of nucleotide sequences, the Clustal Omega (http://www.ebi.ac.uk/Tools/msa/clustalo/) and the BLAST algorithm from the GenBank database (http://blast.ncbi.nlm.nih.gov/Blast.cgi) were used. The sequences were deposited in GenBank under the accession numbers MH734536-MH734603.

The phylogenetic trees were constructed using MEGA 7 software (https://www.megasoftware.net/). Sequences were aligned using MAFFT online alignment tool (https://mafft.cbrc.jp/alignment/server/index.html). The only type material sequences from GenBank were used for phylogenetic trees construction. Strain identification was based upon phylogenetic analysis and similarity of the sequences with GenBank data.

### 2.6. Physiological characteristic of isolates

Physiological properties of the isolated strains were studied by inoculation in 96-well microplates with liquid PYG or CM medium (depending on which media the strain was isolated) supplemented with triphenyltetrazolium chloride (1%) as an indicator of metabolic activity.

Phosphate (KH_2_PO_4_ + H_3_PO_4_ final concentration of 100 mM, pH 2–6) and Tris (Tris + HCl + NaOH final concentration of 100 mM, pH 7–12) buffer systems were used to determine the range of pH values suitable for growth [47]. Tenfold buffer solutions were prepared with pH-meter (Starter 2100, OHAUS Corporation, USA) control. After autoclaving, the buffer solutions were aseptically added to the media. The media pH values were controlled after buffer addition and had no significant changes. The analysis of bacterial pH-resistance was performed at room temperature.

The resistance to the presence of salts was estimated using media with the addition of NaCl, KCl, MgSO_4_, or NaHCO_3_ in concentrations of 2, 5, 10, 15, or 20%, and Mg(ClO_4_)_2_ in concentrations of 0.5, 1, 2, 5, 10, or 15%. Analysis was performed at room temperature.

The temperature-based growth limits were studied by incubating the inoculated medium in thermostat at temperatures 2, 4, 10, 25, 37, 45, or 50 °C.

Antibiotic resistance was tested on liquid media containing 100 µg/mL the following antibiotics (Belmed, Russia): Ampicillin (Amp), cephalexin (Ceph), chloramphenicol (Chl), tetracycline (Tet), doxycycline (Dox), kanamycin (Kan), or rifampicin (Rif). Analysis was performed at room temperature.

During all physiological tests the axenic cultures were inoculated in liquid culture in 96-well plates in three replicates and analysed in ten days incubation. At 2 and 4 °C the incubation time was 30 and 60 days respectively. Positive reaction was estimated by visually registered formazan color, which indicates dehydrogenase cultural activity.

### 2.7. Data analysis

Statistical analysis of data was carried out using STATISTICA 8.0 and Microsoft Office Excel 2007. Indices of similarity and biodiversity of microbial communities were calculated according to Chernov and Lysak [48] at genera level of taxonomic affiliation.

## 3. Results

### 3.1. Bacterial abundance in soil samples

**Figure 1.**
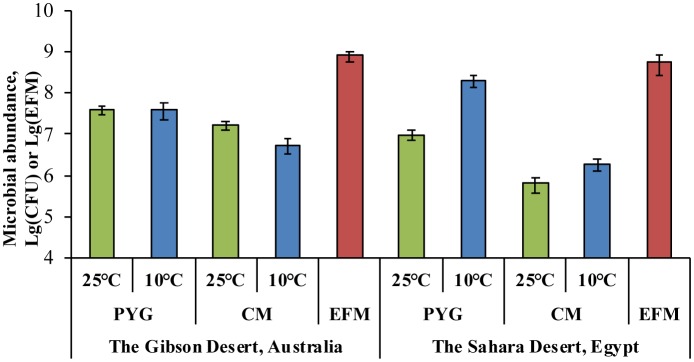
Total prokaryotic cells count obtained by epifluorescent microscopy (EFM) and culturable bacteria count plated on glucose-peptone-yeast (GPY) and Czhapek modified (CM) medias in studied soil samples. Error bars are within standard deviation, *p* < 0.05.

The total number of prokaryotic cells (EFM) was (7.9 ± 2) × 10^8^ cells/g and (5.5 ± 2.8) × 10^8^ cells/g in soil samples from the Gibson Desert and the Sahara Desert, respectively (Figure 1).

The number of heterotrophic aerobic bacteria cultured from the Gibson Desert sample at 25 °C was (4 ± 1) × 10^7^ CFU/g and (1.6 ± 0.4) × 10^7^ CFU/g on GPY medium and CM medium respectively. The number of bacteria cultured at 10 °C was (4 ± 1.7) × 10^7^ CFU/g and (5.6 ± 2.1) × 10^6^ CFU/g on GPY and CM media respectively.

Culturing of the Saharan soil sample at 25 °C revealed (9.8 ± 2.5) × 10^6^ CFU/g and (6.5 ± 2.5) × 10^5^ CFU/g on GPY medium and CM medium, respectively; culturing at 10 °C found (2.0 ± 0.6) × 10^7^ CFU/g and (1.9 ± 0.7) × 10^6^ CFU/g on GPY medium and CM medium, respectively. The formation of bacterial colonies in nutrient media was not observed at 50 °C for 14 days after inoculation.

**Figure 2.**
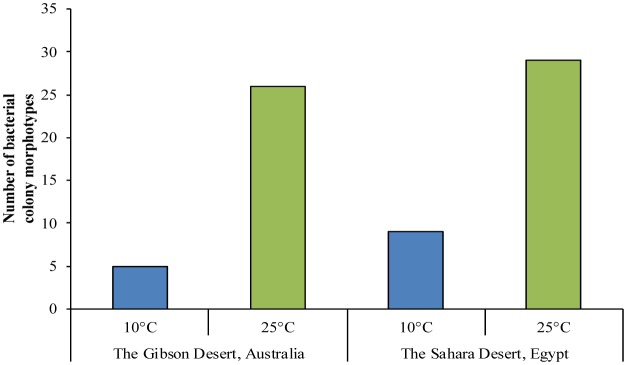
The number of bacterial colonies morphotypes.

The coefficients K = N_total_/CFU_max_ [49] for samples from the Gibson Desert and the Sahara Desert were 19.9 and 2.7 respectively and indicated a high proportion of culturable cells, which are able to reproduce on such medias, in the total cell content of these soils. The data of the diversity of bacterial colony morphotypes on solid media is shown in Figure 2.

The maximum diversity of morphotypes was observed on plates incubated at 25 °C in the Saharan sample (29 morphotypes); in the sample from the Gibson Desert the diversity was slightly lower (26 morphotypes). At 10 °C bacterial communities were less diverse—only 9 morphotypes from the Sahara Desert soil and 5 morphotypes from the Gibson Desert soil were observed. Based on this data, it can be suggested that studied bacterial communities have a mesophilic temperature optimum of growth, but can also operate at lower temperatures. Totally 69 axenic cultures were isolated from both samples: 17 from Gibson Desert plates incubated at 25 °C and 14 from the same sample plates incubated at 10 °C; from Sahara Desert were isolated 29 and 9 strains from plates incubated at 25 and 10 °C respectively.

### 3.2. Potential metabolic activity and functional diversity of microbial community

No MST signals were detected during analysis of native sample from the Gibson Desert. At the same time MST of the sample from the Sahara Desert revealed the intensive utilization of peptone, weak consumption of the lactate, and slight signs of bacterial activity were revealed on glucose, inositol and arabinose.

### 3.3. Taxonomic structure of culturable bacterial communities

The bacterial community isolated from the Gibson Desert was represented by *Actinobacteria* (72%), *Proteobacteria* (15%), *Firmicutes* (12%), and *Bacteroidetes* (1%). In the community isolated at 25 °C the *Aureimonas*, *Micrococcus*, and *Janibacter* (36%, 19%, 19% respectively) representatives were dominant; in the community isolated at 10 °C the representatives of *Brevibacterium* were the most abundant group (56%), in subdominant position were representatives of *Bacillus* genus (22%), and *Micrococcus* and *Paracoccus* genera were constitute 11% of cultivable community each.

The bacterial community isolated from the Sahara Desert was represented by *Actinobacteria* (68%), *Firmicutes* (21%), and *Proteobacteria* (11%). In the community isolated at 25 °C the *Arthrobacter*, *Microvirga*, and *Planomicrobium* (22%, 21%, 20%, respectively) were the most abundant genera; in the community isolated at 10 °C the *Arthrobacter* (41%) and *Massilia* (29%) dominated. Detailed information about culturable community structure is presented in Figure S3. Biodiversity indices of culturable bacterial communities from the Gibson and Sahara Deserts are presented in Table 1.

**Table 1.** Biodiversity indices of investigated culturable communities.

Index	The Gibson desert culturable bacterial community	The Saharan desert culturable bacterial community
Shannon index	3.15	3.12
Evenness index	0.77	0.80
Jaccard index	0.14	
Chekanovskiy-Sørensen index	0.25	
Sørensen modified index	0.20	

Shannon indices of both communities are close and show moderate biodiversity level. However, according to Jaccard, Chekanovskiy-Sørensen, and Sørensen modified indices the communities are characterised by low similarity. The following fact had drawn our attention: The phyla levels of communities are practically similar, but the genera levels are quite different.

Amongst the bacterial community from the Gibson Desert cultured at 10 °C the representatives of the *Bacillus*, *Brevibacillus*, *Brevibacterium*, *Glutamicibacter*, *Micrococcus*, *Paracoccus*, *Pseudochrobactrum*, *Rhodococcus*, *Sphingobacterium* (*S. mizutaii*), and *Streptomyces* genera were identified (Table S1, Figures S1 and S2).

Taxonomic diversity of the bacterial community cultured at 25 °C was much higher. *Agrococcus citreus*, *Agrococcus jenensis*, *Aureimonas altamirensis*, *Microbacterium aurantiacum*, *Microbacterium aurantiacum*, *Microbacterium oxydans*, and *Paenibacillus glucanolyticus* species, as well as representatives of the genera *Bacillus*, *Brevundimonas*, *Janibacter*, *Microbacterium*, *Micrococcus*, and *Rhodococcus* were revealed.

The Saharan Desert microbial community isolated at 10 °C contained representatives of the genera *Artrobacter* (*A. agilis*), *Bacillus*, *Leucobacter*, *Massilia*, *Micrococcus*, *Pseudarthrobacter*, and *Stenotrophomonas*.

In the bacterial community isolated at 25 °C from the same sample, *Arthrobacter agilis*, *Arthrobacter crystallopoietes*, *Bacillus psychrosaccharolyticus*, *Micrococcus cohnii*, *Micrococcus luteus*, *Microvirga soli*, *Paenibacillus glucanolyticus Planomicrobium okeanokoites*, *Pseudomonas putida*, and representatives of the genera *Arthrobacter*, *Bacillus*, *Dietzia*, *Kocuria*, *Microbacterium*, *Micrococcus*, *Planomicrobium*, *Pseudarthrobacter*, *Sphingopyxis*, and *Streptomyces* were identified.

Phylogenetic trees are presented in Figures S3–S5.

### 3.4. Physiological characteristics of bacterial isolates and bacterial communities

The list of studied stress-factors includes environmental influences, widespread in biosphere, which differ in their nature and in their influence on the cells' structures [14],[38]. Moreover, since the deserts are considered in astrobiology as terrestrial analogs of some putative extraterrestrial ecosystems, the study of the list of such stresses could be applied as solution for some astrobiological tasks. In particular, it is hypothesized that presence of water-soluble salts, including perchlorates and sulfates in the Martian regolith, can limit the potential viability of microorganisms on the red planet [50].

Physiological characterization was performed for all 69 strains isolated from both samples (Table S3) in three replicas.

#### 3.4.1. Temperature-based growth limits

**Figure 3.**
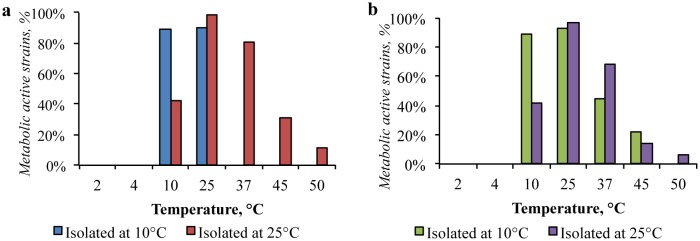
Temperature ranges suitable for growth of: (a) the Gibson Desert strains; (b) the Sahara Desert strains.

The bacterial community isolated from the Gibson Desert at 10 °C was characterized by psychrotolerant temperature range of metabolic activity (T_max_ = 25 °C), and the community isolated at 25 °C implemented a mesophilic growth strategy with an optimum of 25 °C and was metabolically active at the temperature range between 10 °C and 50 °C (Figure 3).

In the same way, cultured bacterial communities of the Sahara Desert were divided into two groups in relation to temperature. The community isolated at 10 °C was metabolically active at the temperatures from 10 °C to 45 °C with an optimum between 10 °C and 25 °C. As for community isolated at 25 °C, the growth in nutrient media at the temperatures between 10 °C and 50 °C with an optimum at 25 °C was detected. These bacterial communities can be characterized as psychrotolerant and mesophilic, respectively.

#### 3.4.2. pH-based growth limits

**Figure 4.**
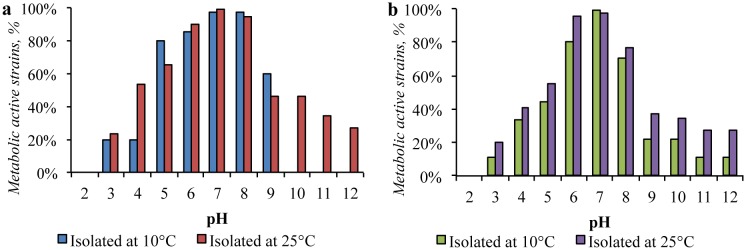
pH ranges suitable for growth of: (a) the Gibson Desert strains; (b) the Sahara Desert strains.

The optimum pH conditions of both bacterial communities isolated at 10 °C and 25 °C from the Gibson Desert were at pH 7–8 (Figure 4). The community isolated at 10 °C was characterized by narrower growth range and by smaller proportion of resistant strains—metabolic activity was observed from pH 3 to pH 9. The community isolated at 25 °C was characterized by broader limits of metabolic activity and by higher proportion of resistant strains—metabolic activity occurred at pH from 3 to 12. The limits of metabolic activity found in both communities can be characterized as tolerant moderately alkalitolerant.

The profiles of resistance to pH values in both bacterial communities isolated from the Sahara Desert were quite similar. Metabolic activity was observed at pH from 3 to 12 with an optimum at pH 7, but higher proportion of resistant strains were noted for the community isolated at 25 °C. This type of community growth strategy can be characterized as tolerant neutrophilic.

#### 3.4.3. Bacteria tolerance to the presence of different salts in culture media

**Figure 5.**
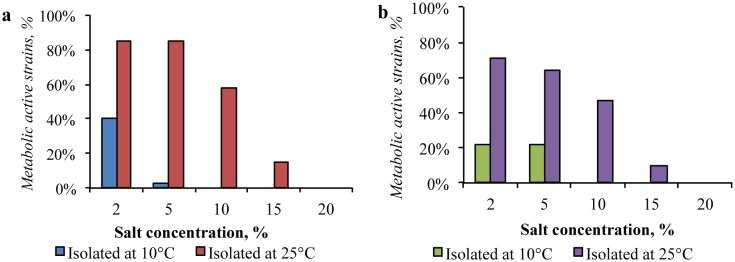
Impact of sodium chloride presence in the media on growth of: (a) the Gibson Desert strains; (b) the Sahara Desert strains.

**Figure 6.**
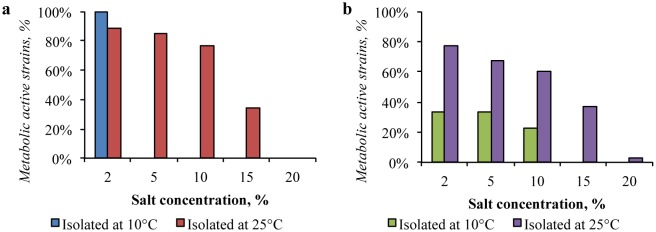
Impact of potassium chloride presence in the media on growth of: (a) the Gibson Desert strains; (b) the Sahara Desert strains.

A high resistance to the presence of sodium and potassium chlorides in the medium (up to 15% of NaCl and KCl) was observed in representatives of the Gibson Desert bacterial community isolated at 25 °C, while the strains isolated at 10 °C metabolized mainly with NaCl and KCl concentrations up to 2% (Figures 5 and 6). Consequently, the community isolated at 25 °C was predominantly moderately-halotolerant, whereas the other community was slightly halotolerant.

In the same way, strains isolated from the Sahara Desert at 25 °C were more resistant to the presence of sodium and potassium chlorides, than the ones isolated at 10 °C. Several strains isolated from this sample could reproduce in media containing 15% of NaCl and 20% of KCl.

The obtained tolerance characteristics allows us to state, that the community isolated at 25 °C was also moderately halotolerant, whereas the one isolated at 10 °C was slightly halotolerant.

According to the proportion of resistant strains in the communities, the Sahara Desert soil bacterial communities were less resistant, than communities, cultured from the Gibson Desert samples.

In addition, the potassium chloride had lower inhibitory effect (from 1.5 to 2.5 times less, depending on concentration) for communities from both samples.

**Figure 7.**
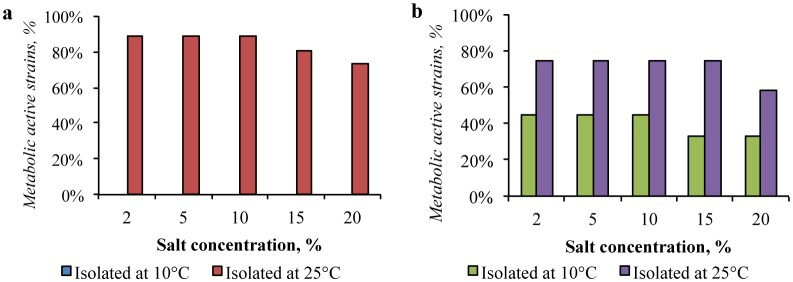
Impact of magnesium sulfate presence in the media on growth of: (a) the Gibson Desert strains; (b) the Sahara Desert strains.

The 88% of strains obtained from the Gibson Desert at 25 °C were capable to metabolize in the presence of up to 10% of MgSO_4_. In the medium containing 20% of this salt, most of the isolates (73%) retained metabolic activity (Figure 7). The resistance to the presence of magnesium sulfate in the representatives of the Gibson Desert bacterial community isolated at 10 °C was not detected.

Similarly, isolates from the Sahara Desert were little inhibited by high concentrations of magnesium sulfate: 58% and 33% of the strains, isolated at 25 °C and 10 °C respectively, tolerated the presence of 20% of the salt.

**Figure 8.**
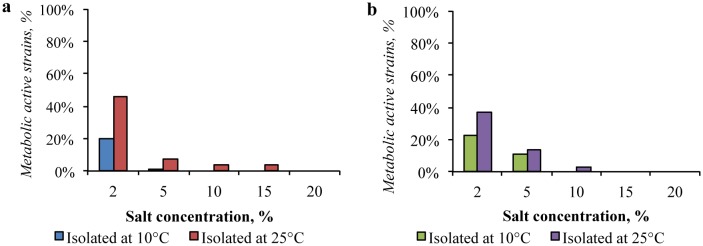
Impact of sodium bicarbonate presence in the media on growth of: (a) the Gibson Desert strains; (b) the Sahara Desert strains.

The presence of sodium bicarbonate significantly inhibited the strains isolated from the both deserts. More than 50% of the strains, isolated from the Gibson Desert at 25 °C were inhibited by the presence of 2% this salt, while only 4% of the isolates retained metabolic activity in the medium containing 15% of this salt (Figure 8). Strains, isolated from the same sample at 10 °C showed even lower resistance to NaHCO_3_. 80% of the strains were not proliferated in the medium, containing 2% of sodium bicarbonate and the growth of all strains was inhibited in the medium containing 5% of this salt. In the same way, the presence of NaHCO_3_ affected strains isolated from the Sahara Desert: 3% of bacteria, isolated at 25 °C, were resistant to the presence of 10% of NaHCO_3_, and 11% of isolates, obtained at 10 °C, tolerated the presence of 5% of this salt.

**Figure 9.**
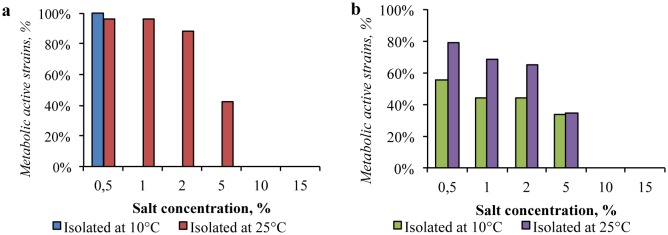
Impact of magnesium perchlorate presence in the media on growth of: (a) the Gibson Desert strains; (b) the Sahara Desert strains.

Significant part of the strains, isolated from the Gibson Desert at 25 °C, was resistant to the presence of magnesium perchlorate. The 88% of the strains retained metabolic activity in the medium containing 2% of Mg(ClO_4_)_2_. Considerable inhibition was registered in the medium containing 5% of this salt: 42% of the strains were metabolically active (Figure 9). Besides, the representatives of the community, obtained at 10 °C, were not culturable with the presence of 1% perchlorate.

The strains isolated from the Sahara Desert also showed high resistance to the presence of magnesium perchlorate: 34% of the all isolates obtained from this sample were able to metabolize in the presence of 5% of the perchlorate, whereas the metabolic activity was not registered with the presence of 10% of this salt. Similar to the tolerance to all previously considered factors, the strains isolated at 10 °C were characterized by lower stress resistance. However, in contrast to the Gibson Desert strains, the 33% of the isolates were able to metabolize in presence of Mg(ClO_4_)_2_ up to 5%.

Detailed information about the tolerance of each strain to all the factors studied is presented in Table S3.

#### 3.4.4. Antibiotic resistance

**Figure 10.**
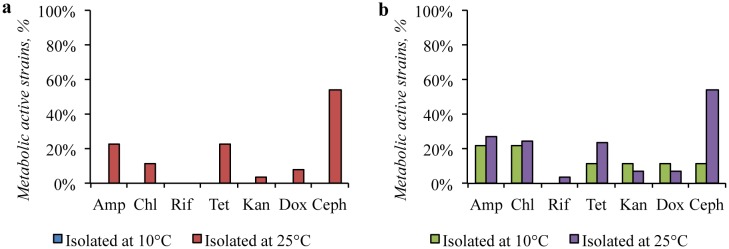
Antibiotic resistance of: (a) the Gibson Desert strains; (b) the Sahara Desert strains.

Among the bacteria, isolated from the Gibson Desert at 10 °C, the antibiotic-resistant strains were not found (Figure 10). Strains, isolated from this sample at 25 °C demonstrated the antibiotic-resistant properties: More than 50% were resistant to cephalexin and 23% were resistant to tetracycline or ampicillin. Among the strains, obtained at 25 °C, there several strains resistant to two or three antibiotics (Table S4) were detected: the strain KBP.AS.15 (*Bacillus* sp.) was resistant to four antibiotics: ampicillin, tetracycline, doxycycline, and cephalexin.

In contrast, among the strains, isolated from the soil of the Sahara Desert at low temperature, the antibiotic-resistance were detected: 20% of the isolates were resistant to ampicillin or chloramphenicol each. In general, these strains were resistant to one antibiotic, however two multidrug-resistant strains were also revealed: the KBP.AS.187 strain (*Shingomonas* sp.) was sensitive only to rifampicin and cephalexin, the KBP.AS.190 strain (*Leucobacter* sp.) was resistant to chloramphenicol and cephalexin.

Among isolates obtained at 25 °C the most common resistance was found to cephalexin (54%), ampicillin (27%), and tetracycline (24%) (details in Table S4). The strain KBP.AS.47 (*Sphingopyxis* sp.) was resistant to presence of all studied antibiotics and the strain KBP.AS.50 (*Arthrobacter* sp.) was resistant to ampicillin, chloramphenicol, kanamycin, and cephalexin.

## 4. Discussion

### 4.1. Bacterial abundance of the soil samples

The obtained values of the total number of prokaryotes and the cultured bacteria count are indicators of high soil enrichment with bacterial cells. Besides, the coefficients K (equal to 19.9 and 2.7 for the Gibson Desert and the Sahara Desert, respectively) indicate a high proportion of culturable bacteria in both microbial communities. These values are consistent with already known literature data on microbial abundance and cultivability of bacteria isolated from arid soils [13],[16],[51],[52] and could be interpreted as an evidence of a shallow metabolic dormancy of these bacteria and the possible metabolic activity of these communities in situ.

The close values of the culturable bacterial number at 25 °C and 10 °C on PYG media (and even equal numbers of cultivated bacteria from the Gibson Desert soil) allow us to assume the existence of wide temperature diapason of community representatives' metabolic activity. The psychrotolerance of desert strains can be explained by significant temperature fluctuations of the environment during the diurnal and seasonal cycles in situ.

Also, it was previously shown that low temperatures lead to increase of oxidative stress level in bacterial cells and, as a result, to the activation of physiological resistance systems to oxidative stress [53]. At the same time, it was showed that desiccation, the presence of water-soluble salts, and high insolation cause oxidative stress in the cell [28],[54], hence the community members are exposed in situ to a high levels of oxidative stress and probably have a number of adaptations to it.

We have found no data on high percentage of psychrotrophic and psychrotolerant bacteria in bacterial communities of desert soils. However, several pure cultures, isolated from such ecotopes are able to reproduce at low temperatures [19],[55]. Moreover, previously it was found that strains isolated from Negev desert soil are able to reproduce at low temperatures - at least down to 2 °C [39]. It also confirms the hypothesis about high resistance of desert bacteria to oxidative stress.

We think that the higher number of culturable cells in the Gibson Desert soil in comparison to the Sahara Desert soil is caused by the climatic regime of these deserts. In the Sahara Desert there is a wider amplitude of temperature fluctuations and lower quantity of atmospheric precipitation, in contrast to the Gibson desert [15].

The observed high number of CFUs and the different structure of culturable community on the CM medium are indicates the presence of bacteria, which are able to assimilate diverse substrates of plant origin requiring pre-hydrolysis (starch and/or sucrose). This result allow us to assume the functional diversity of the microbial community: The culturable community consists of hydrolytic part (who are able to reproduce on CM media) and the part, that does not have their own hydrolases. Such bacteria depends on easily accessible nutrients in the media (such as peptone and yeast extract in PYG media), or metabolites and hydrolysis products of the hydrolytic part. In addition, this result suggests the need of more detailed study on the ability of isolated strains to produce various hydrolytic enzymes, some of which may have industrially significant properties [5].

The absence of bacterial growth at 50 °C allows us to state, that among the cultivated bacteria of both communities there are only facultative thermophilic forms, which is also confirmed by the temperature growth ranges of pure cultures. For pure cultures isolated from desert ecosystems, it was previously shown that the upper temperature growth limit is mainly at 45 °C [56],[57]. However, few individual pure cultures were capable of proliferation at 50 °C. Probably, this occurred due to culturing conditions. The estimation of the number of culturable bacteria in the soils was carried out on agar media, while the study of the physiological parameters of isolated strains was performed in liquid medium.

### 4.2. Taxonomic structure of cultured bacterial communities

The isolated strains from Gibson Desert belong to *Actinobacteria* (72%), *Proteobacteria* (15%), *Firmicutes* (12%), and *Bacteroidetes* (1%). The strains isolated from Sahara Desert belong to *Actinobacteria* (68%), *Firmicutes* (21%), and *Proteobacteria* (11%). Such structure of the community is typical for arid ecosystems [13],[18].

The representatives of genera *Arthrobacter*, *Aureimonas*, *Bacillus*, *Brevibacterium*, *Brevundimonas*, *Dietzia*, *Janibacter*, *Kocuria*, *Massilia*, *Microbacterium*, *Microvirga*, *Paenibacillus*, *Planomicrobium*, *Pseudomonas*, *Pseudarthrobacter*, *Rhodococcus*, *Sphingomonas*, and *Streptomyces* have been earlier isolated from various desert ecosystems [23],[58]–[60].

The representatives of *Microbacteriaceae* family are typical soil bacteria [61]. Nevertheless, we did not find any data in literature about isolation of *Microbacterium aurantiacum* from desert ecosystems. *Dietzia cinnamea*, related to widely distributed *Rhodococcus* genus [62], also appears to be isolated from the desert soil of the Sahara Desert for the first time.

It should be mentioned, that phylogenetic analysis of nucleotide sequences obtained for strains KBP.AS.1 and KBP.AS.3 (*Micrococcus* sp.), KBP.AS.9 and KBP.AS.10 (*Microbacterium aurantiacum*), KBP.AS.20 and KBP.AS.22 (*Microvirga soli*), KBP.AS.32 and KBP.AS.33 (*Planomicrobium* sp.) as well as for strains KBP.AS.127 and KBP.AS.131 (*Rhodococcus* sp.) the 100% similarity occurred, while the strains had clear differences in colony morphology (data not shown) and physiology (Tables S3 and S4). This indicates that these strains are the intrapopulation variation, considering the data showed, that strains from natural habitats have high dissociation activity under stress conditions [63].

Bacteria of the genera *Brevibacterium* and *Paracoccus* isolated from various soil samples are known for their high tolerance to various stress factors [4],[28],[64],[65]. *Agrococcus* *jenensis* isolated from the soil was characterized by moderate halotolerance and antibiotic resistance [66]. For the representatives of the genus *Microbacterium*, the multiple stress tolerance is shown [60]. Among the bacteria of the genus *Paenibacillus*, the halotolerant forms [67],[68] were described and were previously isolated, in particular, from the soils of the Sahara Desert [69]. Among *Arthrobacter*, genera the strains resistant to extreme desiccation [70] to antibiotics, heavy metals, as well as high UV- and radioresistance are known [28],[71]–[74]. Radioresistant and multidrug-resistant forms are found in *Acinetobacter* genera [75]. Among the representatives of the *Bacillus* genus, a variety of extremoloterant forms are known, many of which were isolated from arid ecotopes [69]. For the representatives of genus *Brevundimonas* resistance to heavy metals and ionizing radiation are shown [71],[76],[77]. The resistance of *Janibacter* to alkaline conditions in the medium and to high concentrations of salts was described [78]. Tolerance of *Massilia* to low temperatures [79], high salinity and alkaline pH [80] is proved. Among *Micrococcus* various extremotolerant forms were revealed [81],[82], including those that can persist for a long time in the cryoarid conditions of permafrost [83]. Among the representatives of the *Rhodococcus*, the multiple stress tolerance and the ability to withstand extreme drying was demonstrated [27],[84]. *Streptomyces* are widely known for their high stress tolerance and resistance to antibiotics [31],[85], as well as for the ability to produce antibiotics themselves [86]. Bacterial strains of the genera *Kocuria*, *Planomicrobium*, *Sphingomonas* were previously isolated from a variety of extreme habitats and their resistance to the presence of heavy metals, salts [87]–[89] and to ionizing radiation [28],[90]–[92] was shown. For *Leucobacter* high resistance to chromium and moderate halotolerence was proved [93].

Summing up, taxonomic composition of studied bacterial communities is represented by species and genera common for soils, and is in good agreement with the data obtained for similar samples [94],[95]. Most of the strains, isolated in the current study, are related to genera and species that were previously isolated from various arid ecosystems, or to taxon, for which the stress tolerance was previously shown. We have not found any literature data on the isolation of *Microbacterium aurantiacum* and *Dietzia cinnamea* from arid soils. Thus, we can conclude, that in this study we've isolated this species from arid soil for the first time. Moreover, both communities are characterized by quite similar phylums' structure, represented, meanwhile, by different genera.

### 4.3. Multisubstrate testing

The most intensive utilization of peptone and weak consumption of other carbon sources by microbial community from the Sahara Desert and absence of detectable metabolic activity of the Gibson desert microbial community at MST analysis suggests that the communities needs some growth factors such as vitamins or amino acids, that are present in the peptone, in addition to the sole carbon source. Besides, such results could be caused by specific trophic interactions such as co-metabolism [96], which could be depressed due to pure substances used, sample dilutions, etc.

A similar hypothesis was previously advanced in the study of bacteria in the soil of the Atacama Desert [17]. Moreover, intensive utilization of peptone by the Sahara Desert microbial community can be explained by a larger number of assimilating populations, for example, by those assimilated individual amino acids. In this case, their cumulative metabolic activity is sufficient for its detection.

The consumption of lactose by microbial community indirectly indicates the presence of enterobacterium in the community, which is confirmed by the arabinose utilization [61] also. At the same time, enterobacterium was not found in the cultured bacterial community. It can be explained by the presence of enterobacterium as minor component and by the suppression of their growth by other community representatives. Previous studies also revealed various pathogenic and conditionally pathogenic bacteria in the desert ecosystems [59],[97].

### 4.4. Physiological characteristics of bacterial isolates and bacterial communities

According to the results of physiological testing of bacteria, isolated from the both samples, it was found that bacterial communities isolated at 25 °C are characterized by higher stress tolerance to a diverse stress factors, compared to the isolates obtained from the same samples at lower temperature (10 °C). It can occur due to statistical reasons: Number of strains isolated at 10 °C compared to 25 °C is lower, which could affect comparison results. However, during the diurnal and seasonal cycles the impact of stress factors on bacterial communities is changing, that is why it is possible that during periods of metabolic activity of psychrotolerant bacterial communities *in situ* they are exposed to lower diversity of stress factors' impacts.

The wide temperature ranges of bacteria composing the communities, obtained in vitro as well as the presence of species, whose resistance to desiccation was previously shown (references herein, and [84],[98],[99]) allow us to assume the high adaptability of the studied bacterial communities to their habitats and the possibility of metabolic activity preservation for the most part of the year.

Predominantly alkalitolerant strategy for both studied communities correlate well with the available data on soil bacteria [11] as well as with pH of the corresponding samples. The extension of the metabolic activity range can be interpreted as a consequence of wide fluctuations of external conditions. These fluctuations are causing the unstable pH regime of desert soils because of changes of the solubility of various substances as a result of temperature changes and the contrast mode of precipitation-evaporation processes during the diurnal and annual cycles occur. The predominantly acidotolerant characteristic of the Gibson Desert bacterial community isolated at 10 °C could be due to the limited number of the strains studied.

Despite the fact that the samples we studied are characterized by low content of water-soluble salts [15], we have found weak and moderate halotolerant properties of isolates. It could occur as a result of microbial cells inhabiting micro volumes and, therefore, heterophase systems such as soil. They exist in the conditions of high water-soluble compound concentrations [11], where, in its turn, all soluble molecules are concentrated as a result of colloid interactions [100]. Previous studies of pure bacterial cultures isolated from desert soils showed the presence of several halotolerant strains, which were able to grow at extreme values of the environmental salinity [19],[23],[101]. Besides, all examined salts could be formed in situ during weathering processes [102].

We also should mention high tolerance of all studied bacterial communities to the presence of magnesium sulfate. The presence of this salt slightly inhibits bacterial communities probably because of salt assimilation as an element of mineral nutrition [11]. Previously it was shown that the halotolerant bacteria from the Great Salt Plains (GSP) of Oklahoma are capable of growing at even higher concentrations of MgSO_4_ (up to the 2 M or 24% solutions) [103].

The bacterial communities of the deserts studied were rather slightly inhibited by perchlorate. The higher resistance of isolates from the Gibson Desert is probably associated with higher insolation of this region compared to investigated region of the Sahara Desert [104] and, consequently, with higher level of UV on the surface soil layers, that elevates the level of oxidative stress in bacteria [105]. To date, it is known that the presence of perchlorates in the environment causes oxidative stress to bacterial cells [106]. In general, higher values of halotolerance and stability at low temperatures of isolates from the Gibson Desert show their greater adaptation to oxidative stress [107], which is also confirmed by the high proportion of species in the community that are resistant to desiccation, that also causes oxidative stress [28],[54],[108] and quite similar numbers of cultivated cell on plates incubated at low and at room temperature.

High resistance of both bacterial communities to presence of perchlorates could be associated with high levels or UV-radiation and desiccation in situ as an environmental agents provoking oxidative stress, to which investigated communities are adapted. These results also have an astrobiological implication because of perchlorates and sulfates, that were found in regolith of Mars and are considered to be the factors limiting viability of terrestrial microorganisms in the Martian regolith [48],[109]. Our results show, that terrestrial bacterial communities are well adapted to much higher concentrations of these salts than those found on the Mars [110]. Consequently, terrestrial-like bacteria viability should not be restricted by the presence of sulfates and perchlorates in the regolith.

Among studied factors, sodium bicarbonate had the maximum inhibitory effect on the bacteria. Addition of NaHCO_3_ into the media leads to increase pH of the media and concentration of Na^+^-ions. This inhibition caused by the cytotoxicity of sodium and the increase of membrane permeability in alkaline conditions [111], which promotes active transport of sodium into cytoplasm. Probably, most of the bacteria in the communities studied do not have protective mechanisms for active removal of sodium from the intracellular space and, therefore, are sensitive even to low concentrations of sodium bicarbonate. At the same time, it is possible that NaHCO_3_-resistant forms are minor components in these communities and that is why they have not been found during culturing. In general, tolerance to the sodium bicarbonate is not widespread among soil heterotrophic bacteria [85],[112].

As it was mentioned above, the resistance to various antibiotics has been shown for the genera and species composing studied communities. The antibiotic resistance was higher in the bacterial community from the Sahara Desert, which is probably due to the more contrasting climatic regime of this community [15] and to larger numbers of the strains studied.

We believe that one of the reasons of the distribution of antibiotic-resistant bacterial forms in less-anthropogenically transformed ecosystems is the aggravation of competitive relationships between microbial populations during periods of favorable environmental conditions. Consequently, strains, which are capable of producing antagonistic substances, that suppress competitors, have an advantage in the competition for the substrate. Possibly, the increase of the proportion of producers of antibiotics over time within the community is the driving force for the selection of resistant bacteria in the community. Previous studies have shown the presence of antibiotic producers in desert soils [6],[31]. Consequently, in the periods of active bacterial metabolism in situ the synthesis of antibiotics and subsequent formation of drug-resistant sub-populations is possible.

High proportion of culturable bacteria in the microbial communities testifies in favor of scenario described. It can be assumed that the metabolically inactive part of the community is in a state of shallow metabolic dormancy and is capable to fast reactivation into metabolic-active state under favorable conditions [113], which ultimately leads to aggravation of competitive relationships. At the same time, it is possible that antibiotics in these communities play role of signal metabolites, rather than antagonist substances [114], which can also explain the presence of resistant forms. Both of these hypotheses are supported by the detection of strains resistant to several antibiotics in the studied communities. Multiple antibiotic resistance was found in strains KBP.AS.47 (*Sphingopyxis* sp.), KBP.AS.50 (*Arthrobacter* sp.), KBP.AS.187 (*Shingomonas* sp.), KBP.AS.190 (*Leucobacter aridicolis*), KBP.AS.15 (*Bacillus* sp.) (Table S4).

An extremely important issue is the possibility of multi-resistant strains transferring from the surface of the soil by various natural forces to populated areas or to agricultural production sites. Due to possible horizontal gene transfer in bacterial populations [115], the determinants of multiple resistance can spread among other bacteria [116], in particular, among human's pathogenic, thereby creating danger to people lives in these regions.

## 5. Conclusions

The presented study contributes to the understanding of the structure and functioning of the cultivable bacterial communities of the deserts. A high bacterial abundance in the desert soils was found and significant proportion of culturable cells was revealed. For the first time the abundance of bacteria culturable under low-temperature conditions was assessed for hot desert ecotopes. A close and even equal numbers of psychrotolerant (psychroactive) and mesophilic bacteria in arid ecosystems were revealed.

The taxonomic composition of the culturable bacterial communities of the northeastern Sahara Desert and of the central part of the Gibson Desert is similar to the known data on the other desert ecotopes. Representatives of *Actinobacteria* phylum (among which a lot of extremotolerant species are known) are dominant in the both microbial communities. Bacterial communities showed functional differentiation, which indicates their sustainability, and are characterized by moderate biodiversity.

Our study of the desert-strains resistance to a wide range of various stress factors is unique. The data available in the literature on the tolerance of isolates studied and related species do not contradict our data obtained. Pure bacterial cultures isolated from arid desert ecotopes are characterized by polyextremotolerance and the ability to maintain metabolic activity in vitro in a wide range of physicochemical and biotic influences. We consider that it is caused by the wide-amplitude fluctuations of environmental conditions, which are powerful microevolutionary agents and lead to a physiological adaptation of the representatives of microbial communities, thereby increasing their stress resistance. Probably, the main mechanism of this adaptation process is stress-induced dissociation of community members into morphologically and physiologically diverse strains within one type and further selection of those forms that are stable in changing conditions of desert surface soils. Extrapolating these indicators of resistance to in situ conditions, the year-round activity of these communities and, therefore, participation in biosphere processes can be assumed.

The study of the isolated bacteria ability to produce antagonistic substances, an assessment of the ability to reproduce in nitrogen-free and dilute media and in conditions of water deficiency are in plans of further research, as well as the isolation and study of minor and pathogenic components of the microbial communities.
