# Supplementary material for: Soil bacterial communities of Sahara and Gibson deserts: Physiological and taxonomical characteristics
Source: AIMS Microbiol. 2018 Dec 17;4(4):685–710. doi: 10.3934/microbiol.2018.4.685 (PMC6613332; doi:10.3934/microbiol.2018.4.685)
Supplement: Supplementary file 1 [file microbiol-04-04-685-s001.pdf]

AIMS Microbiology, 4(4): 685–710.
DOI: 10.3934/microbiol.2018.4.685
Received: 22 August 2018
Accepted: 10 December 2018
Published: 17 December 2018

http://www.aimspress.com/journal/microbiology

Research article

Soil bacterial communities of Sahara and Gibson deserts: Physiological and taxonomical characteristics

Andrey A. Belov[1,]*, Vladimir S. Cheptsov[1,2] and Elena A. Vorobyova[1,2]

[1] Soil Science Faculty, Lomonosov Moscow State University, Moscow, Russia
[2] Space Research Institute, Russian Academy of Sciences, Moscow, Russia

* **Correspondence:** Email: and.ant.be@gmail.com; Tel: +79175844407.

Supplementary

Table S1. Taxonomic affiliation of isolated strains.

Sample, temperature of culturing	Strain	Isolation media	GenBank acession number	The most closely related sequences in GenBank	Taxonomic affiliation
Gibson Desert, Australia, 25 ℃	KBP.AS.1	GPY	*MH734536*	*Micrococcus aloeverae KY622923*—99.7% *Micrococcus yunnanensis MH298518*—99.7% *Micrococcus yunnanensis KY622921*—99.5%	*Micrococcus* sp.
Gibson Desert, Australia, 25 ℃	KBP.AS.2	GPY	*MH734568*	*Janibacter indicus MF948904*—100% *Janibacter melonis NR_025805*—100% *Janibacter anophelis NR_043218*—100%	*Janibacter* sp.

Continued on next page

Sample, temperature of culturing	Strain	Isolation media	GenBank acession number	The most closely related sequences in GenBank	Taxonomic affiliation
Gibson Desert, Australia, 25 ℃	KBP.AS.3	GPY	*MH734578*	*Micrococcus aloeverae KY622923—99.7%*<br>*Micrococcus yunnanensis MH298518—99.7%*<br>*Micrococcus yunnanensis KY622921—99.5%*	*Micrococcus* sp.
Gibson Desert, Australia, 25 ℃	KBP.AS.4	GPY	*MH734587*	*Microbacterium oxydans KX369591—99.8%*<br>*Microbacterium oxydans KF975411—99.8%*<br>*Microbacterium oxydans AJ717356—99.8%*	*Microbacterium oxydans*
Gibson Desert, Australia, 25 ℃	KBP.AS.5	GPY	*MH734597*	*Agrococcus sp. KP238419—99.9%*<br>*Agrococcus jenensis NR_026275—99.9%*<br>*Agrococcus citreus NR_041542—99.6%*	*Agrococcus* sp.
Gibson Desert, Australia, 25 ℃	KBP.AS.6	GPY	*MH734599*	*Bacillus pumilus JQ353824—99.5%*<br>*Bacillus safensis MH160088—99.5%*<br>*Bacillus safensis MH671851—99.5%*	*Bacillus* sp.
Gibson Desert, Australia, 25 ℃	KBP.AS.7	GPY	MH734600	*Rhodococcus sp. KF441597—99.8%*<br>*Uncultured Rhodococcus sp. KF504096—99.8%*<br>*Rhodococcus sp. FR772123—99.8%*	*Rhodococcus* sp.
Gibson Desert, Australia, 25 ℃	KBP.AS.8	GPY	*MH734601*	*Bacillus safensis MG645269—100%*<br>*Bacillus pumilus LT906438—100%*<br>*Bacillus zhangzhouensis NR_148786—100%*	*Bacillus* sp.

Continued on next page

Sample, temperature of culturing	Strain	Isolation media	GenBank acession number	The most closely related sequences in GenBank	Taxonomic affiliation
Gibson Desert, Australia, 25 ℃	KBP.AS.9	GPY	*MH734602*	*Microbacterium aurantiacum NR_116476*—99.6 %<br>*Microbacterium aurantiacum NR_116476*—99.6%<br>*Microbacterium kitamiense NR_037048*—99.3%	*Microbacterium aurantiacum*
Gibson Desert, Australia, 25 ℃	KBP.AS.10	GPY	*MH734538*	*Microbacterium aurantiacum NR_116476*—99.6 %<br>*Microbacterium aurantiacum NR_116476*—99.6%<br>*Microbacterium kitamiense NR_037048*—99.3%	*Microbacterium aurantiacum*
Gibson Desert, Australia, 25 ℃	KBP.AS.11	GPY	*MH734539*	*Aurantimonas altamirensis EU442517*—99.9%<br>*Aureimonas altamirensis NR_043764*—99.8%<br>*EU442517*—99.9%<br>*Aureimonas altamirensis NR_043764*—99.8%	*Aureimonas altamirensis*
Gibson Desert, Australia, 25 ℃	KBP.AS.12	GPY	*MH734541*	*Microbacterium lacus* KX082876—99.7%<br>*Microbacterium aurum* KJ127516—99.5%<br>*Microbacterium lacus* HM209729 – 99.1%	*Microbacterium* sp.
Gibson Desert, Australia, 25 ℃	KBP.AS.13	GPY	*MH734548*	*Brevundimonas vesicularis MG966304*—99.9%<br>*Brevundimonas vesicularis NR_113586*—99.5%<br>*Brevundimonas nasdae MG650163*—99.9%	*Brevundimonas* sp.
Gibson Desert, Australia, 25 ℃	KBP.AS.14	GPY	*MH734551*	*Paenibacillus glucanolyticus KC789782*—99.9%<br>*Paenibacillus glucanolyticus CP015286*—99.7%<br>*Paenibacillus glucanolyticus NR_113748*—99.6%	*Paenibacillus glucanolyticus*

Continued on next page

Sample, temperature of culturing	Strain	Isolation media	GenBank acession number	The most closely related sequences in GenBank	Taxonomic affiliation
Gibson Desert, Australia, 25 ℃	KBP.AS.15	GPY	*MH734552*	*Bacillus drentensis NR_118438—99.6%*<br>*Bacillus infantis NR_043267—99.4%*<br>*Bacillus infantis MH130045—99.6%*	*Bacillus* sp.
Gibson Desert, Australia, 25 ℃	KBP.AS.16	GPY	*MH734553*	*Agrococcus sp. KC160778.1—100%*<br>*Agrococcus sp. EU584505—100%*<br>*Agrococcus citreus NR_041542—100%*	*Agrococcus citreus*
Gibson Desert, Australia, 25 ℃	KBP.AS.18	GPY	*MH734559*	*Bacillus subtilis NR_116187—99.9%*<br>*Bacillus subtilis CP019663—99.6%*<br>*Bacillus tequilensis MH010390—99.8%*	*Bacillus* sp.
Sahara Desert, Egypt, 25 ℃	KBP.AS.19	GPY	*MH734566*	*Bacillus litoralis MF101139—100%*<br>*Bacillus niabensis KC788148 —100%*<br>*Bacillus halosaccharovorans NR_109116—99.7%*	*Bacillus* sp.
Sahara Desert, Egypt, 25 ℃	KBP.AS.20	GPY	*MH734569*	*Microvirga sp. KX444133—99.1%*<br>*Microvirga soli KX247636—99.1%*<br>*Microvirga aerilata NR_114298—98.0%*	*Microvirga soli*
Sahara Desert, Egypt, 25 ℃	KBP.AS.21	GPY	*MH734570*	*Arthrobacter sp. JX949321—97.8%*<br>*Arthrobacter agilis JQ684255.1—97.6%*<br>*Arthrobacter sp. KC986992—97.4%*	*Arthrobacter* sp.

Continued on next page

Sample, temperature of culturing	Strain	Isolation media	GenBank acession number	The most closely related sequences in GenBank	Taxonomic affiliation
Sahara Desert, Egypt, 25 ℃	KBP.AS.22	GPY	*MH734571*	*Microvirga sp. KX444133—99.4%* *Microvirga soli KX247636—99.4%* *Microvirga soli KX247636—99.4%*	*Microvirga soli*
Sahara Desert, Egypt, 25 ℃	KBP.AS.23	GPY	*MH734572*	*Planomicrobium okeanokoites HQ848119—99.8%* *Planomicrobium okeanokoites HQ848112—99.8%*	*Planomicrobium okeanokoites*
Sahara Desert, Egypt, 25 ℃	KBP.AS.24	GPY	*MH734573*	*Pseudarthrobacter phenanthrenivorans NR_074770—99.6%* *Pseudarthrobacter phenanthrenivorans CP002379—99.6%* *Arthrobacter phenanthrenivorans NR_042469—98.5%*	*Pseudarthrobacter* sp.
Sahara Desert, Egypt, 25 ℃	KBP.AS.25	GPY	*MH734574*	*Arthrobacter sp. KJ191025—99.8%* *Arthrobacter parietis JF505951—99.7%* *Arthrobacter parietis NR_042252—99.7%*	*Arthrobacter* sp.
Sahara Desert, Egypt, 25 ℃	KBP.AS.26	GPY	*MH734575*	*Paenibacillus glucanolyticus CP028366—99.5%* *Paenibacillus glucanolyticus MG051308—99.5%* *Paenibacillus glucanolyticus MG051307—99.5%*	*Paenibacillus glucanolyticus*
Sahara Desert, Egypt, 25 ℃	KBP.AS.27	GPY	*None*	*Was identified using morpho-phisiological method.*	*Bacillus* sp.
Sahara Desert, Egypt, 25 ℃	KBP.AS.28	GPY	*MH734576*	*Streptomyces sp. KM067288—97.6%* *Uncultured actinobacterium clone H127 GQ504259—97.6%* *Streptomyces sp. EU847137—97.6%*	*Streptomyces* sp.

Continued on next page

Sample, temperature of culturing	Strain	Isolation media	GenBank acession number	The most closely related sequences in GenBank	Taxonomic affiliation
Sahara Desert, Egypt, 25 ℃	KBP.AS.29	GPY	*MH734577*	*Arthrobacter agilis KC788154—99.5%*<br>*Arthrobacter agilis KC788153—99.5%*<br>*Arthrobacter agilis KC788098—99.5%*	*Arthrobacter agilis*
Sahara Desert, Egypt, 25 ℃	KBP.AS.30	GPY	*MH734579*	*Bacillus psychrosaccharolyticus MF101012—99.2%*<br>*Bacillus psychrosaccharolyticus NR_113992—99.1%*<br>*Bacillus muralis NR_042083—96.3%*	*Bacillus psychrosaccharolyticus*
Sahara Desert, Egypt, 25 ℃	KBP.AS.31	GPY	*MH734580*	*Kocuria polaris HG515399—99.7%*<br>*Kocuria himachalensis LC113906—99.0%*	*Kocuria* sp.
Sahara Desert, Egypt, 25 ℃	KBP.AS.32	GPY	*MH734581*	*Planomicrobium sp. KU951464.1—99.4%*<br>*Planomicrobium sp. MF041832.1—99.1%*<br>*Planomicrobium chinense KC842235—98.9%*	*Planomicrobium* sp.
Sahara Desert, Egypt, 25 ℃	KBP.AS.33	GPY	*MH734582*	*Planomicrobium stackebrandtii LC076757—99.5%*<br>*Planomicrobium soli NR_134133.1—99.7%*<br>*Planomicrobium okeanokoites NR_113593—99.2%*	*Planomicrobium* sp.
Sahara Desert, Egypt, 25 ℃	KBP.AS.34	GPY	*MH734537*	*Arthrobacter sp. KM507593—99.7%*<br>*Arthrobacter sp. JX949321.2—99.7%*<br>*Arthrobacter sp. JX949321.2—99.6%*	*Arthrobacter* sp.

Continued on next page

Sample, temperature of culturing	Strain	Isolation media	GenBank acession number	The most closely related sequences in GenBank	Taxonomic affiliation
Sahara Desert, Egypt, 25 ℃	KBP.AS.35	GPY	*MH734583*	*Dietzia cinnamea NR_116686*—100%<br>*Dietzia papillomatosis NR_116687*—99.9%<br>*Dietzia lutea CP015449*—98.9%	*Dietzia sp.*
Sahara Desert, Egypt, 25 ℃	KBP.AS.36	GPY	*MH734584*	*Micrococcus aloeverae NR_134088*—100%<br>*Micrococcus luteus LS483396*—99.8%<br>*Micrococcus endophyticus KY933306*—99.8%	*Micrococcus* sp.
Sahara Desert, Egypt, 25 ℃	KBP.AS.37	GPY	*MH734585*	*Kocuria assamensis KT989850*—99.9%<br>*Kocuria palustris NR_026451*—99.9%<br>*Kocuria assamensis NR_132604*—99.7%	*Kocuria* sp.
Sahara Desert, Egypt, 25 ℃	KBP.AS.38	GPY	*MH734586*	*Micrococcus luteus JN545040*—99.5%<br>*Micrococcus luteus MH211276*—99.1%<br>*Micrococcus sp. MG214549*—99.1%	*Micrococcus* sp.
Sahara Desert, Egypt, 25 ℃	KBP.AS.41	CM	*MH734588*	*Micrococcus cohnii NR_117194*—100%<br>*Micrococcus cohnii KP974711*—100%<br>*Micrococcus sp. GQ169060*—100%	*Micrococcus cohnii*
Sahara Desert, Egypt, 25 ℃	KBP.AS.42	CM	*MH734589*	*Arthrobacter crystallopoietes CP018864*—99.8%<br>*Arthrobacter crystallopoietes JN393316*—99.8%<br>*Arthrobacter crystallopoietes KU285616*—99.8%	*Arthrobacter crystallopoietes*

Continued on next page

Sample, temperature of culturing	Strain	Isolation media	GenBank acession number	The most closely related sequences in GenBank	Taxonomic affiliation
Sahara Desert, Egypt, 25 ℃	KBP.AS.43	CM	*MH734590*	*Arthrobacter crystallopoietes KC778371*—99.9% *Arthrobacter crystallopoietes CP018864*—99.8% *Arthrobacter globiformis NR_112192*—97.6%	*Arthrobacter crystallopoietes*
Sahara Desert, Egypt, 25 ℃	KBP.AS.44	CM	*MH734591*	*Bacillus safensis MG645269*—100% *Bacillus pumilus LT906438*—100% *Bacillus zhangzhouensis NR_148786*—100%	*Bacillus* sp.
Sahara Desert, Egypt, 25 ℃	KBP.AS.45	CM	*MH734592*	*Microbacterium nematophilum KF499505*—98,9% *Microbacterium sp. MG719566*—98,8% *Microbacterium kyungheense MF373498*—98,9%	*Microbacterium* sp.
Sahara Desert, Egypt, 25 ℃	KBP.AS.46	CM	*MH734593*	*Pseudomonas putida MG846038*—99.8% *Pseudomonas putida MH636786*—99.5% *Pseudomonas putida MH620727*—99.5%	*Pseudomonas putida*
Sahara Desert, Egypt, 25 ℃	KBP.AS.47	CM	*MH734595*	*Sphingopyxis chilensis KY393080*—99.7% *Sphingopyxis panaciterrae NR_112561*—98.6% *Sphingopyxis chilensis NR_024631*—99.1%	*Sphingopyxis* sp.
Sahara Desert, Egypt, 25 ℃	KBP.AS.48	CM	*MH734596*	*Bacillus subtilis HQ111353*—99.9% *Bacillus velezensis KY694464*—99.9%	*Bacillus* sp.
Sahara Desert, Egypt, 25 ℃	KBP.AS.50	CM	*MH734598*	*Pseudomonas putida MG846038*—99.8% *Pseudomonas putida MH580215*—99.3% *Pseudomonas putida MH580208*—99.3%	*Pseudomonas putida*

Continued on next page

Sample, temperature of culturing	Strain	Isolation media	GenBank acession number	The most closely related sequences in GenBank	Taxonomic affiliation
Gibson Desert, Australia, 10 ℃	KBP.AS.119	CM	*MH734540*	*Streptomyces sp.* KX573860—99.6% *Streptomyces caniferus* NR_116636—99.5% *Streptomyces glebosus* NR_117951—99.4%	*Streptomyces* sp.
Gibson Desert, Australia, 10 ℃	KBP.AS.120	CM	*MH734542*	*Glutamicibacter nicotianae MG813752—99.5% Glutamicibacter nicotianae MG813750—99.5% Glutamicibacter arilaitensis MG788347—99.5%*	*Glutamicibacter* sp.
Gibson Desert, Australia, 10 ℃	KBP.AS.123	CM	*MH734543*	*Brevibacillus agri KY818990—100% Brevibacillus brevis KJ782629—100% Brevibacillus reuszeri NR_113802—99.3%*	*Brevibacillus* sp.
Gibson Desert, Australia, 10 ℃	KBP.AS.125	CM	*MH734544*	*Bacillus subtilis KX281183.1—99.4% Bacillus subtilis KR999950.1—99.3% Bacillus subtilis KR233009.1—99.1%*	*Bacillus* sp.
Gibson Desert, Australia, 10 ℃	KBP.AS.127	CM	MH734545	*Rhodococcus fascians MH605375—98.5% Rhodococcus cerastii MF777046—98.5% Rhodococcus fascians MF974596—98.5%*	*Rhodococcus* sp.
Gibson Desert, Australia, 10 ℃	KBP.AS.128	CM	*MH734546*	*Pseudochrobactrum asaccharolyticum NR_042474.1—100% Pseudochrobactrum kiredjianiae NR_042519—100%*	*Pseudochrobactrum* sp.
Gibson Desert, Australia, 10 ℃	KBP.AS.129	CM	*MH734547*	*Sphingobacterium mizutaii LT906468—99.0% Sphingobacterium mizutaii MF179537—99.0%*	*Sphingobacterium mizutaii*

Continued on next page

Sample, temperature of culturing	Strain	Isolation media	GenBank acession number	The most closely related sequences in GenBank	Taxonomic affiliation
Gibson Desert, Australia, 10 ℃	KBP.AS.131	CM	MH734549	*Rhodococcus fascians MH605375*—99.0% *Rhodococcus cerastii MF777046*—99.0% *Rhodococcus sp. MF664191*—99.0%	*Rhodococcus* sp.
Gibson Desert, Australia, 10 ℃	KBP.AS.132	CM	MH734550	*Bacillus aryabhattai MG430234*—98.5% *Bacillus sp. KC236676*—98.5% *Bacillus megaterium JQ831622*—98.5%	*Bacillus* sp.
Gibson Desert, Australia, 10 ℃	KBP.AS.174	GPY	*MH734554*	*Brevibacterium epidermidis KY992553*—99.7% *Brevibacterium sediminis NR_153678*—99.7% *Brevibacterium siliguriense LT629766*—99.6%	*Brevibacterium* sp.
Gibson Desert, Australia, 10 ℃	KBP.AS.175	GPY	*MH734555*	*Brevibacterium sp. HQ202806*—100% *Brevibacterium sp. DQ448693*—100% *Brevibacterium sp. MG819325*—99,8%	*Brevibacterium* sp.
Gibson Desert, Australia, 10 ℃	KBP.AS.176	GPY	*MH734556*	*Micrococcus sp. JX239759—99.5% Micrococcus sp. AM990848—99.3% Micrococcus luteus MG859501*—99.3%	*Micrococcus* sp.
Gibson Desert, Australia, 10 ℃	KBP.AS.177	GPY	*MH734557*	*Paracoccus sp. MH173273*—100% *Paracoccus carotinifaciens MG988383*—100% *Paracoccus marcusii NR_044922*—100%	*Paracoccus* sp.

Continued on next page

Sample, temperature of culturing	Strain	Isolation media	GenBank acession number	The most closely related sequences in GenBank	Taxonomic affiliation
Gibson Desert, Australia, 10 ℃	KBP.AS.179	GPY	*MH734558*	*Bacillus cereus GQ495663*—100%<br>*Bacillus wiedmannii NR_152692*—100%<br>*Bacillus thuringiensis MG645258*—100%<br>*Bacillus anthracis LC379953*—100%	*Bacillus* sp.
Sahara Desert, Egypt, 10 ℃	KBP.AS.183	GPY	*MH734560*	*Massilia sp. KY635898*—99.3%<br>*Massilia suwonensis NR_116872*—99.0%<br>*Massilia niabensis NR_044571*—98.8%	*Massilia* sp.
Sahara Desert, Egypt, 10 ℃	KBP.AS.184	GPY	MH734561	*Micrococcus* sp. *KT583428*—100%<br>Micrococcus sp. *FJ457288*—99.8%<br>*Micrococcus* sp. *FJ015031*—99.8%	*Micrococcus* sp.
Sahara Desert, Egypt, 10 ℃	KBP.AS.185	GPY	*MH734562*	*Arthrobacter agilis KR085874.* – 99.0%<br>*Arthrobacter agilis JQ825269.1*—99.0%<br>*Arthrobacter agilis KC788152*—99.0%	*Artrobacter agilis*
Sahara Desert, Egypt, 10 ℃	KBP.AS.186	GPY	*MH734603*	*Micrococcus sp. JX239759*—99.5%<br>*Micrococcus sp. AM990848*—99.3%<br>*Micrococcus aloeverae MH553939*—99.3%	*Micrococcus* sp.
Sahara Desert, Egypt, 10 ℃	KBP.AS.187	GPY	*MH734563*	*Leucobacter sp. KY623368*—99.6%<br>*Leucobacter aridicollis KR827428*—99.6%<br>*Leucobacter aridicollis KC764981*—99.6%	*Leucobacter* sp.

Continued on next page

Sample, temperature of culturing	Strain	Isolation media	GenBank acession number	The most closely related sequences in GenBank	Taxonomic affiliation
Sahara Desert, Egypt, 10 ℃	KBP.AS.188	GPY	MH734564	*Stenotrophomonas sp. MH703457—99.0% Stenotrophomonas maltophilia MF536870—99.0% Stenotrophomonas sp. MF774134—99.0%*	*Stenotrophomonas maltophilia*
Sahara Desert, Egypt, 10 ℃	KBP.AS.189	GPY	*MH734565*	*Pseudarthrobacter phenanthrenivorans NR_074770—99.7% Pseudarthrobacter phenanthrenivorans CP002379—99.7% Pseudarthrobacter phenanthrenivorans NR_042469—99.6%*	*Pseudarthrobacter* sp.
Sahara Desert, Egypt, 10 ℃	KBP.AS.190	GPY	*MH734567*	*Leucobacter sp. KP152582—99.3% Leucobacter aridicollis KJ742507—99.3% Leucobacter aridicollis KR827428—99.2%*	*Leucobacter* sp.
Sahara Desert, Egypt, 10 ℃	KBP.AS.465	CM	*MH734594*	*Bacillus licheniformis MG607364—99.7% Bacillus subtilis CP029052—99.6% Bacillus licheniformis MH482980—99.6%*	*Bacillus* sp.

Table S2. The primers used for 16S rRNA gene amplification and sequencing.

GenBank acession number	Strain	Taxonomic affiliation	Primers used for the amplification	Primers used for the sequencing
MH734536	KBP.AS.1	*Micrococcus* sp.	27f + Un1492r	1100r
MH734568	KBP.AS.2	*Janibacter* sp.	27f + Un1492r	1100r
MH734578	KBP.AS.3	*Micrococcus* sp.	27f + Un1492r	1100r
MH734587	KBP.AS.4	*Microbacterium oxydans*	341f + 805r	537r
MH734597	KBP.AS.5	*Agrococcus* sp.	27f + Un1492r	1100r
MH734599	KBP.AS.6	*Bacillus* sp.	341f + 805r	805r
MH734600	KBP.AS.7	*Rhodococcus* sp.	27f + 537r	537r
MH734601	KBP.AS.8	*Bacillus* sp.	27f + Un1492r	1100r

Continued on next page

GenBank acession number	Strain	Taxonomic affiliation	Primers used for the amplification	Primers used for the sequencing
MH734602	KBP.AS.9	*Microbacterium aurantiacum*	27f + Un1492r	1100r
MH734538	KBP.AS.10	*Microbacterium aurantiacum*	27f + Un1492r	1100r
MH734539	KBP.AS.11	*Aureimonas altamirensis*	27f + Un1492r	1100r
MH734541	KBP.AS.12	*Microbacterium* sp.	27f + Un1492r	1100r
MH734548	KBP.AS.13	*Brevundimonas* sp.	27f + Un1492r	1100r
MH734551	KBP.AS.14	*Paenibacillus glucanolyticus*	27f + Un1492r	1100r
MH734552	KBP.AS.15	*Bacillus* sp.	27f + Un1492r	1100r
MH734553	KBP.AS.16	*Agrococcus citreus*	27f + 537r	537r
MH734559	KBP.AS.18	*Bacillus* sp.	27f + Un1492r	1100r
MH734566	KBP.AS.19	*Bacillus* sp.	27f + Un1492r	1100r
MH734569	KBP.AS.20	*Microvirga soli*	27f + Un1492r	1100r
MH734570	KBP.AS.21	*Arthrobacter* sp.	27f + 537r	537r
MH734571	KBP.AS.22	*Microvirga soli*	63f + 1387r	1100r
MH734572	KBP.AS.23	*Planomicrobium okeanokoites*	27f + Un1492r	1100r
MH734573	KBP.AS.24	*Pseudarthrobacter* sp.	27f + Un1492r	1100r
MH734574	KBP.AS.25	*Arthrobacter* sp.	63f + 1387r	1100r
MH734575	KBP.AS.26	*Paenibacillus glucanolyticus*	341f + 805r	805r
None	KBP.AS.27	*Bacillus* sp.	Identified by morphology	
MH734576	KBP.AS.28	*Streptomyces* sp.	341f + 805r	805r
MH734577	KBP.AS.29	*Arthrobacter agilis*	341f + 805r	805r
MH734579	KBP.AS.30	*Bacillus psychrosaccharolyticus*	27f + Un1492r	1100r
MH734580	KBP.AS.31	*Kocuria* sp.	63f + 1387r	1100r
MH734581	KBP.AS.32	*Planomicrobium* sp.	341f + 805r	805r
MH734582	KBP.AS.33	*Planomicrobium* sp.	27f + Un1492r	1100r
MH734537	KBP.AS.34	*Arthrobacter* sp.	63f + 1387r	1100r
MH734583	KBP.AS.35	*Dietzia* sp.	63f + 1387r	1100r
MH734584	KBP.AS.36	*Micrococcus* sp.	63f + 1387r	1100r
MH734585	KBP.AS.37	*Kocuria* sp.	27f + Un1492r	1100r
MH734586	KBP.AS.38	*Micrococcus* sp.	27f + Un1492r	1100r
MH734588	KBP.AS.41	*Micrococcus cohnii*	27f + 537r	537r
MH734589	KBP.AS.42	*Arthrobacter crystallopoietes*	63f + 1387r	537r
MH734590	KBP.AS.43	*Arthrobacter crystallopoietes*	27f + Un1492r	1100r
MH734591	KBP.AS.44	*Bacillus* sp.	27f + Un1492r	1100r
MH734592	KBP.AS.45	*Microbacterium* sp.	27f + Un1492r	1100r
MH734593	KBP.AS.46	*Pseudomonas putida*	341f + 805r	805r
MH734595	KBP.AS.47	*Sphingopyxis* sp.	63f + 1387r	1100r
MH734596	KBP.AS.48	*Bacillus* sp.	27f + Un1492r	1100r
MH734598	KBP.AS.50	*Pseudomonas putida*	341f + 805r	805r
MH734540	KBP.AS.119	*Streptomyces* sp.	63f + 1387r	1100r
MH734542	KBP.AS.120	*Glutamicibacter* sp.	341f + 805r	805r

Continued on next page

GenBank acession number	Strain	Taxonomic affiliation	Primers used for the amplification	Primers used for the sequencing
MH734543	KBP.AS.123	*Brevibacillus* sp.	27f + 537r	537r
MH734544	KBP.AS.125	*Bacillus* sp.	27f + 537r	537r
MH734545	KBP.AS.127	*Rhodococcus* sp.	63f + 1387r	1100r
MH734546	KBP.AS.128	*Pseudochrobactrum* sp.	27f + 537r	537r
MH734547	KBP.AS.129	*Sphingobacterium mizutaii*	27f + 537r	537r
MH734549	KBP.AS.131	*Rhodococcus* sp.	63f + 1387r	1100r
MH734550	KBP.AS.132	*Bacillus* sp.	63f + 1387r	1100r
MH734554	KBP.AS.174	*Brevibacterium* sp.	27f + Un1492r	1100r
MH734555	KBP.AS.175	*Brevibacterium* sp.	27f + 537r	537r
MH734556	KBP.AS.176	*Micrococcus* sp.	341f + 805r	805r
MH734557	KBP.AS.177	*Paracoccus* sp.	27f + Un1492r	805r
MH734558	KBP.AS.179	*Bacillus* sp.	27f + Un1492r	1100r
MH734560	KBP.AS.183	*Massilia* sp.	27f + Un1492r	1100r
MH734561	KBP.AS.184	*Micrococcus* sp.	27f + 537r	537r
MH734562	KBP.AS.185	*Artrobacter agilis*	341f + 805r	805r
MH734603	KBP.AS.186	*Micrococcus* sp.	341f + 805r	805r
MH734563	KBP.AS.187	*Leucobacter* sp.	27f + Un1492r	1100r
MH734564	KBP.AS.188	*Stenotrophomonas maltophilia*	63f + 1387r	1100r
MH734565	KBP.AS.189	*Pseudarthrobacter* sp.	63f + 1387r	1100r
MH734567	KBP.AS.190	*Leucobacter* sp.	27f + Un1492r	1100r
MH734594	KBP.AS.465	*Bacillus* sp.	341f + 805r	805r
Amplification program for 341f + 805r primer pair		Amplification program for 63f + 1387r primer pair	Amplification program for 27f + Un1492r and 27f + 541r primer pairs	
1. 95 ℃ 3:00		1. 4 ℃ 10:00	1. 94 ℃ 3:00	
2. 95 ℃ 0:40		2. 95 ℃ 3:00	2. 55 ℃ 0:30	
3. 55 ℃ 0:40		3. 94 ℃ 0:30	3. 72 ℃ 1:00	
4. 72 ℃ 0:40		4. 60 ℃ 0:30	4. 94 ℃ 0:30	
Go to step 2 4×		5. 72 ℃ 0:45	Go to step 2 5×	
5. 95 ℃ 0:30		Go to step 3 36×	5. 55 ℃ 0:20	
6. 55 ℃ 0:30		6. 72 ℃ 3:00	6. 72 ℃ 0:40	
7. 72 ℃ 0:30			7. 94 ℃ 0:30	
Go to step 6 23×			Go to step 6 32×	
8. 1. 72 ℃ 5:00			8. 55 ℃ 0:30	
			9. 72 ℃ 5:00	

Table S3. Physiological characteristics of isolates.

Strain	Sample, temperature of culturing	Temperature, ℃	pH	Maximum salt concentrations at which growth is possible, %				
				NaCl, %	KCl, %	$MgSO_4$, %	$NaHCO_3$, %	$Mg(ClO_4)_2$, %
KBP.AS.1	Gibson Desert, Australia, 25 ℃	25–37	6–12	10	15	15	15	5
KBP.AS.2	Gibson Desert, Australia, 25 ℃	10–25	6–8	<2	2	<2	<2	5
KBP.AS.3	Gibson Desert, Australia, 25 ℃	25–37	6–12	10	15	20	2	5
KBP.AS.4	Gibson Desert, Australia, 25 ℃	10–25	3–8	<2	<2	<2	<2	5
KBP.AS.5	Gibson Desert, Australia, 25 ℃	25–45	4–10	10	10	20	2	2
KBP.AS.6	Gibson Desert, Australia, 25 ℃	25–37	6–12	10	15	20	2	5
KBP.AS.7	Gibson Desert, Australia, 25 ℃	25–37	5–11	10	15	20	2	5
KBP.AS.8	Gibson Desert, Australia, 25 ℃	10–25	3–8	<2	<2	10	2	5
KBP.AS.9	Gibson Desert, Australia, 25 ℃	25–37	6–12	10	10	20	2	2
KBP.AS.10	Gibson Desert, Australia, 25 ℃	25–37	5–12	10	10	20	5	5
KBP.AS.11	Gibson Desert, Australia, 25 ℃	25–37	6–11	10	10	20	2	5
KBP.AS.12	Gibson Desert, Australia, 25 ℃	25–37	5–12	10	10	20	2	5
KBP.AS.13	Gibson Desert, Australia, 25 ℃	*10–50**	3–8	15	15	20	<2	1
KBP.AS.14	Gibson Desert, Australia, 25 ℃	25–45	3–8	15	15	20	<2	1
KBP.AS.15	Gibson Desert, Australia, 25 ℃	25–50	3–8	15	15	20	<2	2
KBP.AS.16	Gibson Desert, Australia, 25 ℃	25–37	6–12	10	15	15	2	5
KBP.AS.18	Gibson Desert, Australia, 25 ℃	*10–50**	3–8	15	15	20	<2	2
KBP.AS.19	Sahara Desert, Egypt, 25 ℃	25–37	4–8	10	15	20	<2	0,5
KBP.AS.20	Sahara Desert, Egypt, 25 ℃	25–25	3–8	15	15	20	<2	<0,5
KBP.AS.21	Sahara Desert, Egypt, 25 ℃	25–50	3–8	15	15	20	<2	2

Continued on next page

Strain	Sample, temperature of culturing	Temperature, ℃	pH	Maximum salt concentrations at which growth is possible, %				
				NaCl, %	KCl, %	$MgSO_4$, %	$NaHCO_3$, %	$Mg(ClO_4)_2$, %
KBP.AS.22	Sahara Desert, Egypt, 25 ℃	10–37	3–8	15	15	15	<2	1
KBP.AS.23	Sahara Desert, Egypt, 25 ℃	25–37	5–12	10	15	20	2	2
KBP.AS.24	Sahara Desert, Egypt, 25 ℃	25–37	5–12	10	15	20	2	5
KBP.AS.25	Sahara Desert, Egypt, 25 ℃	25–37	6–12	10	15	15	5	5
KBP.AS.26	Sahara Desert, Egypt, 25 ℃	25–37	6–12	10	15	20	2	5
KBP.AS.27	Sahara Desert, Egypt, 25 ℃	25–37	6–12	10	10	15	10	2
KBP.AS.28	Sahara Desert, Egypt, 25 ℃	25–37	6–12	10	15	15	5	5
KBP.AS.29	Sahara Desert, Egypt, 25 ℃	10–25	6–8	<2	2	<2	<2	0,5
KBP.AS.30	Sahara Desert, Egypt, 25 ℃	25–37	6–12	10	15	20	2	5
KBP.AS.31	Sahara Desert, Egypt, 25 ℃	10–25	3–8	<2	2	15	<2	5
KBP.AS.32	Sahara Desert, Egypt, 25 ℃	25–25	6–8	<2	<2	<2	<2	<0,5
KBP.AS.33	Sahara Desert, Egypt, 25 ℃	10–25	5–8	<2	<2	<2	<2	<0,5
KBP.AS.34	Sahara Desert, Egypt, 25 ℃	25–25	6–8	<2	<2	<2	<2	<0,5
KBP.AS.35	Sahara Desert, Egypt, 25 ℃	25–25	6–8	<2	<2	<2	<2	<0,5
KBP.AS.36	Sahara Desert, Egypt, 25 ℃	25–25	6–8	<2	<2	<2	<2	<0,5
KBP.AS.37	Sahara Desert, Egypt, 25 ℃	25–50	3–8	2	5	20	2	5
KBP.AS.38	Sahara Desert, Egypt, 25 ℃	10–25	6–8	<2	<2	<2	<2	5
KBP.AS.41	Sahara Desert, Egypt, 25 ℃	10–37	4–10	10	10	20	2	5
KBP.AS.42	Sahara Desert, Egypt, 25 ℃	10–45	*3–12**	10	20	20	5	2

Continued on next page

Strain	Sample, temperature of culturing	Temperature ℃	pH	Maximum salt concentrations at which growth is possible, %				
				NaCl, %	KCl, %	$MgSO_4$, %	$NaHCO_3$, %	$Mg(ClO_4)_2$, %
KBP.AS.43	Sahara Desert, Egypt, 25 ℃	25–37	6–8	5	10	20	<2	2
KBP.AS.44	Sahara Desert, Egypt, 25 ℃	10–45	4–10	10	10	20	2	5
KBP.AS.45	Sahara Desert, Egypt, 25 ℃	10–37	5–8	5	10	20	<2	2
KBP.AS.46	Sahara Desert, Egypt, 25 ℃	10–37	4–9	2	2	20	<2	0,5
KBP.AS.47	Sahara Desert, Egypt, 25 ℃	10–37	4–9	5	5	20	<2	2
KBP.AS.48	Sahara Desert, Egypt, 25 ℃	25–37	6–8	5	10	20	<2	2
KBP.AS.50	Sahara Desert, Egypt, 25 ℃	10–37	4–8	5	10	20	<2	2
KBP.AS.119	Gibson Desert, Australia, 10 ℃	25–25	6–8	<2	<2	<2	<2	<0,5
KBP.AS.120	Gibson Desert, Australia, 10 ℃	10–37	4–10	5	5	10	2	2
KBP.AS.123	Gibson Desert, Australia, 10 ℃	10–37	4–8	5	10	20	<2	2
KBP.AS.125	Gibson Desert, Australia, 10 ℃	10–45	4–8	5	10	20	<2	2
KBP.AS.127	Gibson Desert, Australia, 10 ℃	25–37	4–8	5	5	20	<2	2
KBP.AS.128	Gibson Desert, Australia, 10 ℃	10–45	4–8	5	10	20	<2	2
KBP.AS.129	Gibson Desert, Australia, 10 ℃	10–37	4–8	5	10	20	<2	2
KBP.AS.131	Gibson Desert, Australia, 10 ℃	10–45	4–8	5	10	20	<2	2
KBP.AS.132	Gibson Desert, Australia, 10 ℃	25	5–10	10	10	20	<2	2
KBP.AS.174	Gibson Desert, Australia, 10 ℃	10–25	5–9	2	2	<2	<2	0,5
KBP.AS.175	Gibson Desert, Australia, 10 ℃	10–25	5–9	2	2	<2	<2	0,5
KBP.AS.176	Gibson Desert, Australia, 10 ℃	10–25	5–9	<2	2	<2	<2	0,5

Continued on next page

Strain	Sample, temperature of culturing	Temperature ℃	pH	Maximum salt concentrations at which growth is possible, %				
				NaCl, %	KCl, %	$MgSO_4$, %	$NaHCO_3$, %	$Mg(ClO_4)_2$, %
KBP.AS.177	Gibson Desert, Australia, 10 ℃	10–25	6–8	<2	2	<2	<2	0,5
KBP.AS.179	Gibson Desert, Australia, 10 ℃	10–25	3–8	<2	2	<2	2	0,5
KBP.AS.183	Sahara Desert, Egypt, 10 ℃	10–25	6–8	<2	<2	<2	<2	<0,5
KBP.AS.184	Sahara Desert, Egypt, 10 ℃	10–25	6–8	<2	<2	<2	<2	<0,5
KBP.AS.185	Sahara Desert, Egypt, 10 ℃	25–37	6–8	<2	<2	10	<2	5
KBP.AS.186	Sahara Desert, Egypt, 10 ℃	10–25	4–8	<2	<2	<2	<2	0,5
KBP.AS.187	Sahara Desert, Egypt, 10 ℃	10–45	5–10	5	10	20	2	5
KBP.AS.188	Sahara Desert, Egypt, 10 ℃	25–37	3–8	<2	5	20	<2	5
KBP.AS.189	Sahara Desert, Egypt, 10 ℃	25–25	6–8	<2	<2	<2	<2	<0,5
KBP.AS.190	Sahara Desert, Egypt, 10 ℃	10–45	*4–12**	5	10	20	5	2
KBP.AS.465	Sahara Desert, Egypt, 10 ℃	25–25	6–8	<2	<2	<2	<2	<0,5

*: Strains characterised with widest temperature or pH ranges of growth.

Table S4. Antibiotic resistance spectra of isolates

Strain	Sample, temperature of culturing	Amp	Chl	Rif	Tet	Kan	Dox	Ceph
KBP.AS.1	Gibson Desert, Australia, 25 ℃	S	R	S	S	S	S	R
KBP.AS.2	Gibson Desert, Australia, 25 ℃	S	S	S	S	S	S	S
KBP.AS.3	Gibson Desert, Australia, 25 ℃	S	S	S	S	S	S	R
KBP.AS.4	Gibson Desert, Australia, 25 ℃	S	S	S	S	S	S	S
KBP.AS.5	Gibson Desert, Australia, 25 ℃	R	S	S	S	S	S	S
KBP.AS.6	Gibson Desert, Australia, 25 ℃	S	S	S	S	S	S	R
KBP.AS.7	Gibson Desert, Australia, 25 ℃	S	S	S	R	S	S	R
KBP.AS.8	Gibson Desert, Australia, 25 ℃	S	S	S	S	S	S	S
KBP.AS.9	Gibson Desert, Australia, 25 ℃	S	S	S	S	S	S	R
KBP.AS.10	Gibson Desert, Australia, 25 ℃	S	R	S	R	S	S	R
KBP.AS.11	Gibson Desert, Australia, 25 ℃	S	S	S	R	S	S	R

Continued on next page

Strain	Sample, temperature of culturing	Amp	Chl	Rif	Tet	Kan	Dox	Ceph
KBP.AS.12	Gibson Desert, Australia, 25 ℃	S	S	S	R	R	S	R
KBP.AS.13	Gibson Desert, Australia, 25 ℃	R	S	S	S	S	S	R
KBP.AS.14	Gibson Desert, Australia, 25 ℃	R	S	S	S	S	R	R
KBP.AS.15	Gibson Desert, Australia, 25 ℃	R	S	S	R	S	R	R
KBP.AS.16	Gibson Desert, Australia, 25 ℃	S	R	S	R	S	S	R
KBP.AS.18	Gibson Desert, Australia, 25 ℃	R	S	S	S	S	S	S
KBP.AS.19	Sahara Desert, Egypt, 25 ℃	S	S	S	S	S	S	R
KBP.AS.20	Sahara Desert, Egypt, 25 ℃	S	S	S	R	S	S	S
KBP.AS.21	Sahara Desert, Egypt, 25 ℃	S	S	S	R	S	R	S
KBP.AS.22	Sahara Desert, Egypt, 25 ℃	R	S	S	S	S	S	R
KBP.AS.23	Sahara Desert, Egypt, 25 ℃	S	R	S	S	S	S	R
KBP.AS.24	Sahara Desert, Egypt, 25 ℃	S	S	S	R	S	S	R
KBP.AS.25	Sahara Desert, Egypt, 25 ℃	S	S	S	S	S	S	R
KBP.AS.26	Sahara Desert, Egypt, 25 ℃	S	R	S	R	S	S	S
KBP.AS.27	Sahara Desert, Egypt, 25 ℃	S	S	S	R	S	S	R
KBP.AS.28	Sahara Desert, Egypt, 25 ℃	S	S	S	S	S	S	R
KBP.AS.29	Sahara Desert, Egypt, 25 ℃	S	S	S	S	S	S	S
KBP.AS.30	Sahara Desert, Egypt, 25 ℃	S	R	S	R	S	S	R
KBP.AS.31	Sahara Desert, Egypt, 25 ℃	S	S	S	S	S	S	S
KBP.AS.32	Sahara Desert, Egypt, 25 ℃	S	S	S	S	S	S	S
KBP.AS.33	Sahara Desert, Egypt, 25 ℃	S	S	S	S	S	S	S
KBP.AS.34	Sahara Desert, Egypt, 25 ℃	S	S	S	S	S	S	S
KBP.AS.35	Sahara Desert, Egypt, 25 ℃	S	S	S	S	S	S	S
KBP.AS.36	Sahara Desert, Egypt, 25 ℃	S	S	S	S	S	S	S
KBP.AS.37	Sahara Desert, Egypt, 25 ℃	R	S	S	S	S	S	S
KBP.AS.38	Sahara Desert, Egypt, 25 ℃	S	S	S	S	S	S	S
KBP.AS.41	Sahara Desert, Egypt, 25 ℃	R	S	S	S	S	S	S
KBP.AS.42	Sahara Desert, Egypt, 25 ℃	S	R	S	S	S	S	R
KBP.AS.43	Sahara Desert, Egypt, 25 ℃	R	S	S	S	S	S	R
KBP.AS.44	Sahara Desert, Egypt, 25 ℃	R	R	S	S	S	S	R
KBP.AS.45	Sahara Desert, Egypt, 25 ℃	S	S	S	S	S	S	R
KBP.AS.46	Sahara Desert, Egypt, 25 ℃	S	S	S	S	S	S	R
KBP.AS.47	Sahara Desert, Egypt, 25 ℃	R	R	R	R	R	R	R
KBP.AS.48	Sahara Desert, Egypt, 25 ℃	R	S	S	S	S	S	R
KBP.AS.50	Sahara Desert, Egypt, 25 ℃	R	R	S	S	R	S	R
KBP.AS.119	Gibson Desert, Australia, 10 ℃	R	R	S	S	S	S	S
KBP.AS.120	Gibson Desert, Australia, 10 ℃	S	S	S	S	S	S	S
KBP.AS.123	Gibson Desert, Australia, 10 ℃	S	S	S	S	S	S	S
KBP.AS.125	Gibson Desert, Australia, 10 ℃	S	S	S	S	S	S	R
KBP.AS.127	Gibson Desert, Australia, 10 ℃	R	S	S	S	S	S	S
KBP.AS.128	Gibson Desert, Australia, 10 ℃	S	S	S	S	S	S	S

Continued on next page

Strain	Sample, temperature of culturing	Amp	Chl	Rif	Tet	Kan	Dox	Ceph
KBP.AS.129	Gibson Desert, Australia, 10 ℃	S	S	S	S	S	S	S
KBP.AS.131	Gibson Desert, Australia, 10 ℃	S	S	S	S	S	S	R
KBP.AS.132	Gibson Desert, Australia, 10 ℃	S	S	S	S	S	S	S
KBP.AS.174	Gibson Desert, Australia, 10 ℃	R	S	S	S	S	S	S
KBP.AS.175	Gibson Desert, Australia, 10 ℃	R	S	S	S	S	S	S
KBP.AS.176	Gibson Desert, Australia, 10 ℃	R	S	S	S	S	S	S
KBP.AS.177	Gibson Desert, Australia, 10 ℃	S	S	S	S	S	S	S
KBP.AS.179	Gibson Desert, Australia, 10 ℃	S	S	S	S	S	S	S
KBP.AS.183	Sahara Desert, Egypt, 10 ℃	S	S	S	S	S	S	S
KBP.AS.184	Sahara Desert, Egypt, 10 ℃	R	S	S	S	S	S	S
KBP.AS.185	Sahara Desert, Egypt, 10 ℃	S	S	S	S	S	S	S
KBP.AS.186	Sahara Desert, Egypt, 10 ℃	S	S	S	S	S	S	S
KBP.AS.187	Sahara Desert, Egypt, 10 ℃	R	R	S	R	R	R	S
KBP.AS.188	Sahara Desert, Egypt, 10 ℃	R	S	S	S	S	S	S
KBP.AS.189	Sahara Desert, Egypt, 10 ℃	R	S	S	S	S	S	S
KBP.AS.190	Sahara Desert, Egypt, 10 ℃	S	R	S	S	S	S	R
KBP.AS.465	Sahara Desert, Egypt, 10 ℃	R	S	S	S	S	S	S

R: Resistant strain; S: Sensitive strain; Amp: Ampicillin (100 mkg/ml); Chl: Chloramphenicol (100 mkg/ml); Rif: Rifampicin (100 mkg/ml); Tet: Tetracycline (100 mkg/ml); Kan: Kanamycin (100 mkg/ml); Dox: Doxycycline (100 mkg/ml); Ceph: Cephalexin (100 mkg/ml).

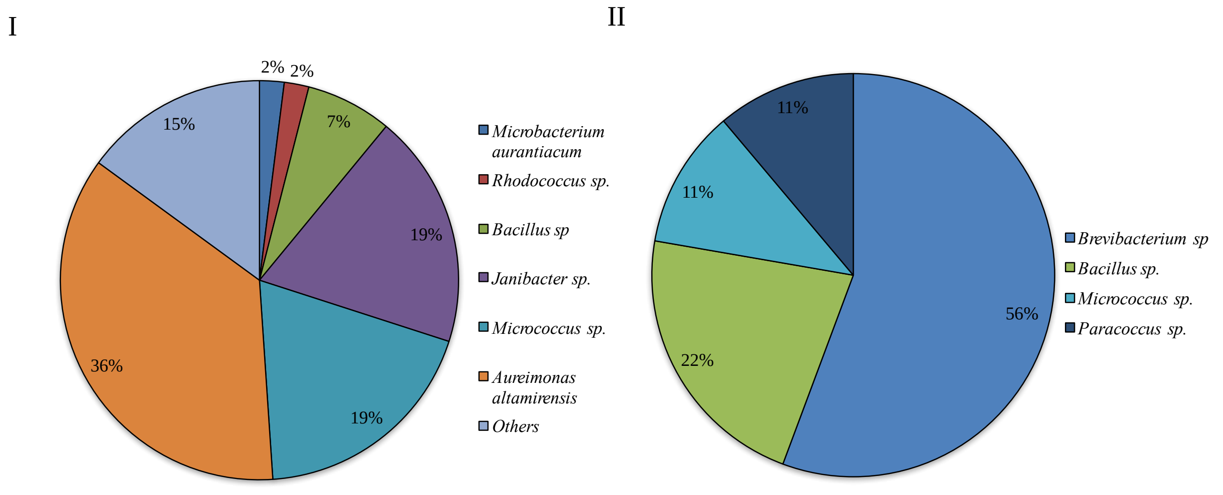

Figure S1. Taxonomic structure of the Gibson desert soil culturable bacterial communities: (I) community isolated at 25 ℃, (II) community isolated at 10 ℃. Each strain of the ones combined in the "others" group accounted for about 1% of the number of cultured bacteria in the community. This group includes *Acinetobacter* sp., *Agrococcus citreus*, *Agrococcus jenensis*, *Bacillus* sp., *Brevibacillus* sp., *Brevundimonas* sp., *Glutamicibacter* sp., *Microbacterium oxidans*, Microbacterium sp., *Paenibacillus glucanolyticus*, *Pseudochrobactrum* sp., *Sphingobacterium mizutaii*, *Streptomyces* sp.

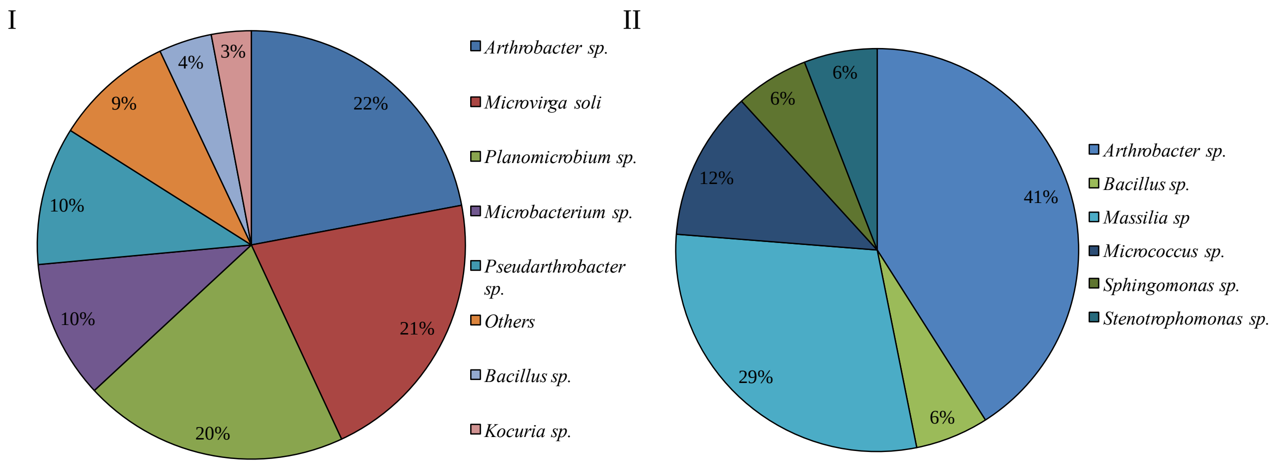

Figure S2. Taxonomic structure of the Sahara desert soil culturable bacterial communities: (I) community isolated at 25 ℃, (II) community isolated at 10 ℃. Each strain of the ones combined in the "others" group accounted for about 1% of the number of cultured bacteria in the community. This group includes *Arthrobacter agilis*, *Arthrobacter crystallopoietes*, *Bacillus psychrosaccharolyticus*, *Dietzia cinnamea*, *Micrococcus cohnii*, *Micrococcus luteus*, *Micrococcus* sp., *Planomicrobium okeanokoites*, *Pseudomonas putida*, *Sphingomonas echinoides*, *Sphingomonas* sp., *Sphingopyxis* sp., *Streptomyces* sp.

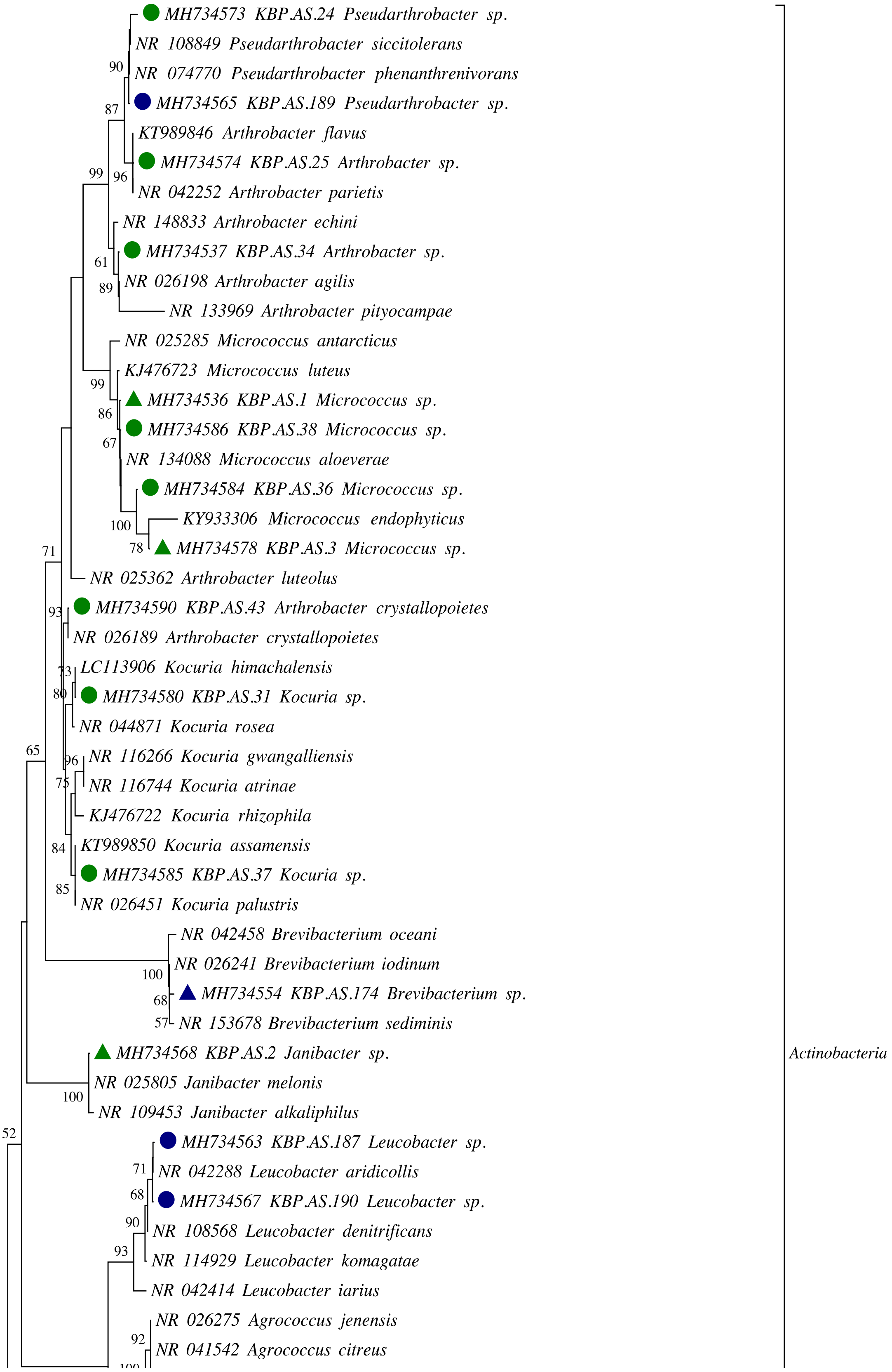
MH734573 KBP.AS.24 Pseudarthrobacter sp.
NR 108849 Pseudarthrobacter siccitolerans
NR 074770 Pseudarthrobacter phenanthrenivorans
MH734565 KBP.AS.189 Pseudarthrobacter sp.
KT989846 Arthrobacter flavus
MH734574 KBP.AS.25 Arthrobacter sp.
NR 042252 Arthrobacter parietis
NR 148833 Arthrobacter echini
MH734537 KBP.AS.34 Arthrobacter sp.
NR 026198 Arthrobacter agilis
NR 133969 Arthrobacter pityocampae
NR 025285 Micrococcus antarcticus
KJ476723 Micrococcus luteus
MH734536 KBP.AS.1 Micrococcus sp.
MH734586 KBP.AS.38 Micrococcus sp.
NR 134088 Micrococcus aloeverae
MH734584 KBP.AS.36 Micrococcus sp.
KY933306 Micrococcus endophyticus
MH734578 KBP.AS.3 Micrococcus sp.
NR 025362 Arthrobacter luteolus
MH734590 KBP.AS.43 Arthrobacter crystallopoietes
NR 026189 Arthrobacter crystallopoietes
LC113906 Kocuria himachalensis
MH734580 KBP.AS.31 Kocuria sp.
NR 044871 Kocuria rosea
NR 116266 Kocuria gwangalliensis
NR 116744 Kocuria atrinae
KJ476722 Kocuria rhizophila
KT989850 Kocuria assamensis
MH734585 KBP.AS.37 Kocuria sp.
NR 026451 Kocuria palustris
NR 042458 Brevibacterium oceani
NR 026241 Brevibacterium iodinum
MH734554 KBP.AS.174 Brevibacterium sp.
NR 153678 Brevibacterium sediminis
MH734568 KBP.AS.2 Janibacter sp.
NR 025805 Janibacter melonis
NR 109453 Janibacter alkaliphilus
MH734563 KBP.AS.187 Leucobacter sp.
NR 042288 Leucobacter aridicollis
MH734567 KBP.AS.190 Leucobacter sp.
NR 108568 Leucobacter denitrificans
NR 114929 Leucobacter komagatae
NR 042414 Leucobacter iarius
NR 026275 Agrococcus jenensis
NR 041542 Agrococcus citreus
Actinobacteria

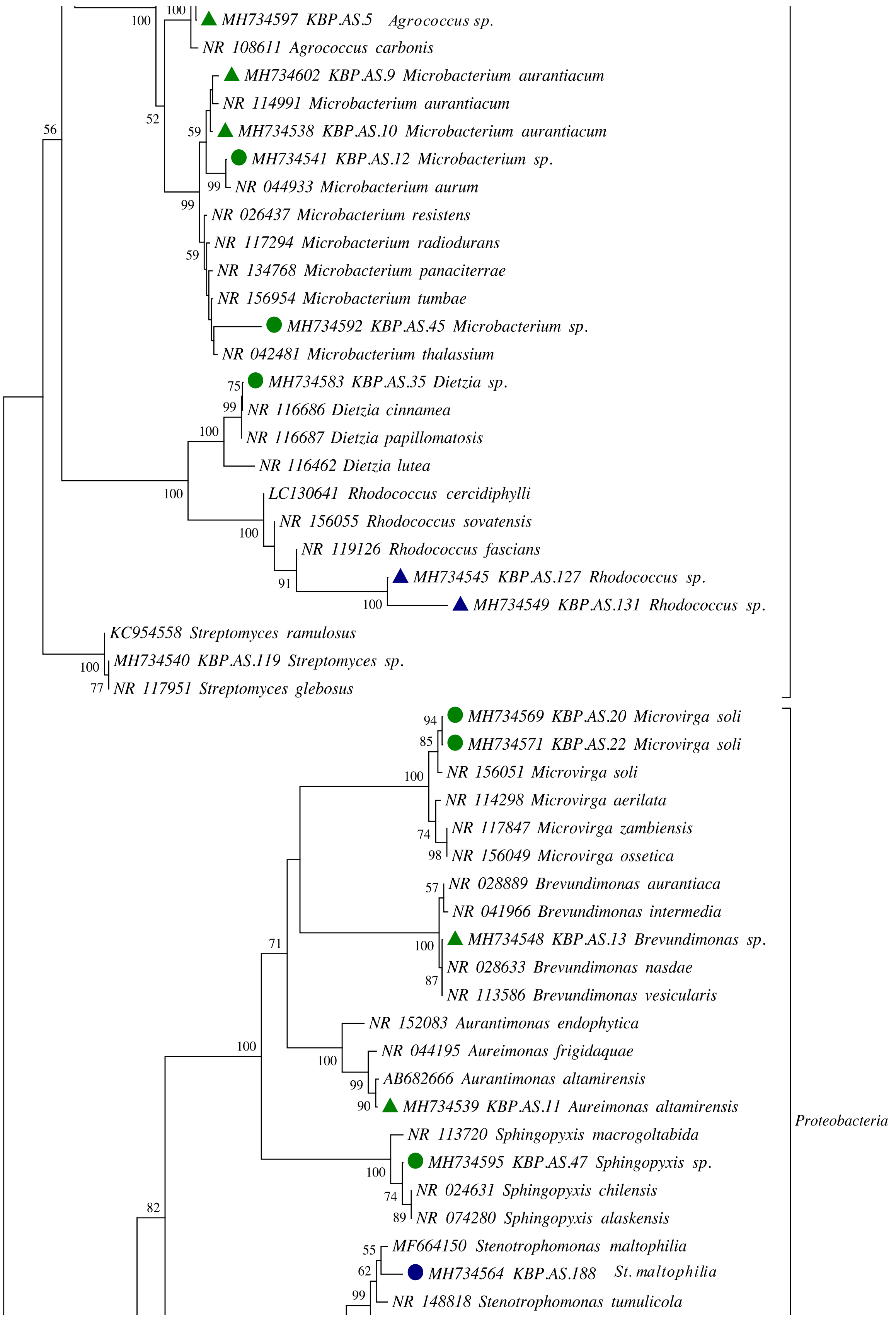
MH734597 KBP.AS.5 Agrococcus sp.
NR 108611 Agrococcus carbonis
MH734602 KBP.AS.9 Microbacterium aurantiacum
NR 114991 Microbacterium aurantiacum
MH734538 KBP.AS.10 Microbacterium aurantiacum
MH734541 KBP.AS.12 Microbacterium sp.
NR 044933 Microbacterium aurum
NR 026437 Microbacterium resistens
NR 117294 Microbacterium radiodurans
NR 134768 Microbacterium panaciterrae
NR 156954 Microbacterium tumbae
MH734592 KBP.AS.45 Microbacterium sp.
NR 042481 Microbacterium thalassium
MH734583 KBP.AS.35 Dietzia sp.
NR 116686 Dietzia cinnamea
NR 116687 Dietzia papillomatosis
NR 116462 Dietzia lutea
LC130641 Rhodococcus cercidiphylli
NR 156055 Rhodococcus sovatensis
NR 119126 Rhodococcus fascians
MH734545 KBP.AS.127 Rhodococcus sp.
MH734549 KBP.AS.131 Rhodococcus sp.
KC954558 Streptomyces ramulosus
MH734540 KBP.AS.119 Streptomyces sp.
NR 117951 Streptomyces glebosus
MH734569 KBP.AS.20 Microvirga soli
MH734571 KBP.AS.22 Microvirga soli
NR 156051 Microvirga soli
NR 114298 Microvirga aerilata
NR 117847 Microvirga zambiensis
NR 156049 Microvirga ossetica
NR 028889 Brevundimonas aurantiaca
NR 041966 Brevundimonas intermedia
MH734548 KBP.AS.13 Brevundimonas sp.
NR 028633 Brevundimonas nasdae
NR 113586 Brevundimonas vesicularis
NR 152083 Aurantimonas endophytica
NR 044195 Aureimonas frigidaquae
AB682666 Aurantimonas altamirensis
MH734539 KBP.AS.11 Aureimonas altamirensis
NR 113720 Sphingopyxis macrogoltabida
MH734595 KBP.AS.47 Sphingopyxis sp.
NR 024631 Sphingopyxis chilensis
NR 074280 Sphingopyxis alaskensis
MF664150 Stenotrophomonas maltophilia
MH734564 KBP.AS.188 St. maltophilia
NR 148818 Stenotrophomonas tumulicola
Proteobacteria

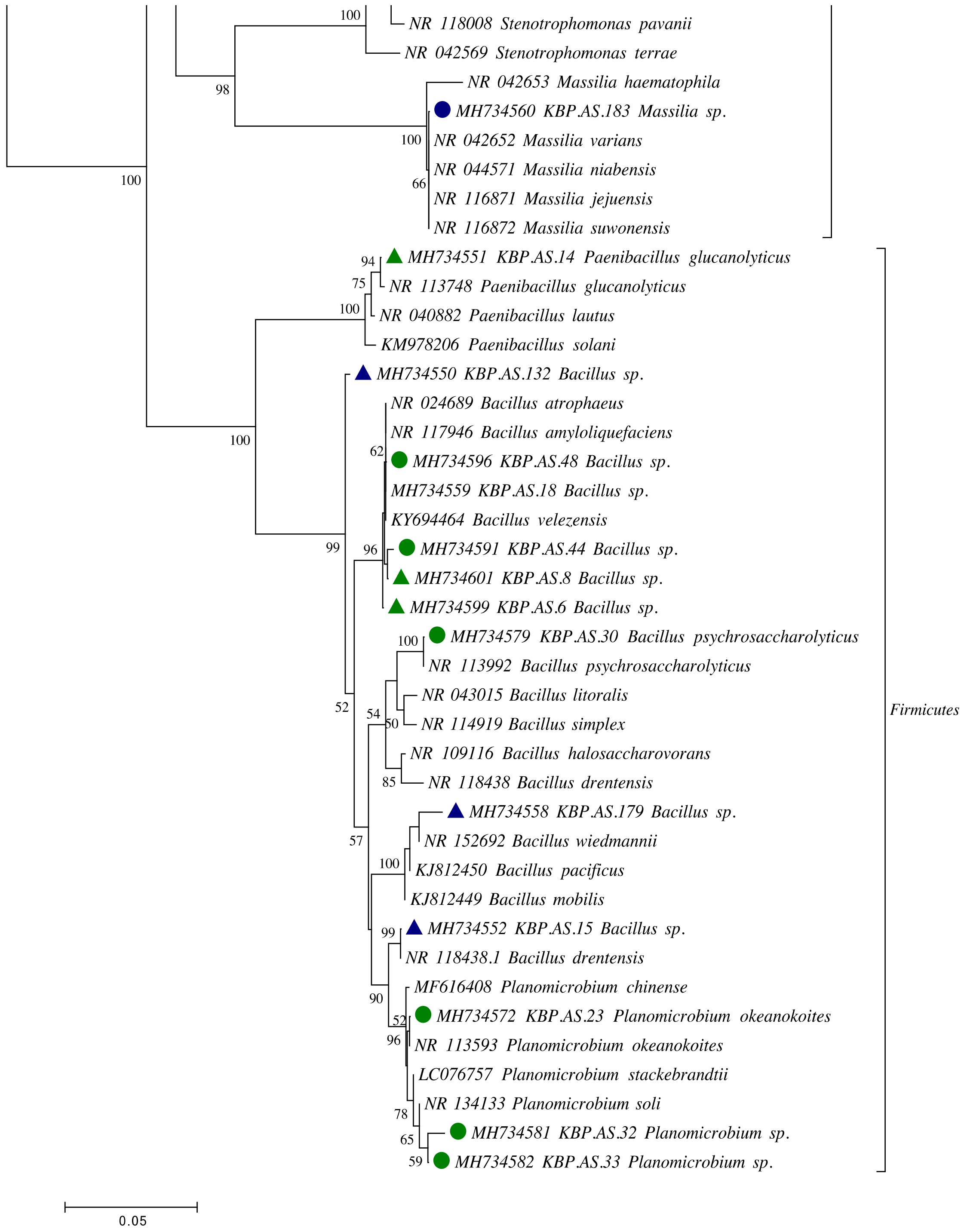

Figure S3. Phylogenetic analysis of 16S rRNA genes of the strains isolated from desert soils which were sequenced using 1100r primer. The tree was constructed using the Neighbor-Joining method with the use of the most closely related sequences from GenBank. The number of taxa—82. The number of significant characters—640. The number of bootstrap replicates—500. Confidence values of branching are given for cases of 50% and higher. Circles indicate strains isolated from the Sahara desert, triangles—the Gibson desert; green and blue colours indicate strains isolated at 25 and 10 ℃, respectively. The scale under the tree indicates the number of phylogenetically significant changes of nucleotides.

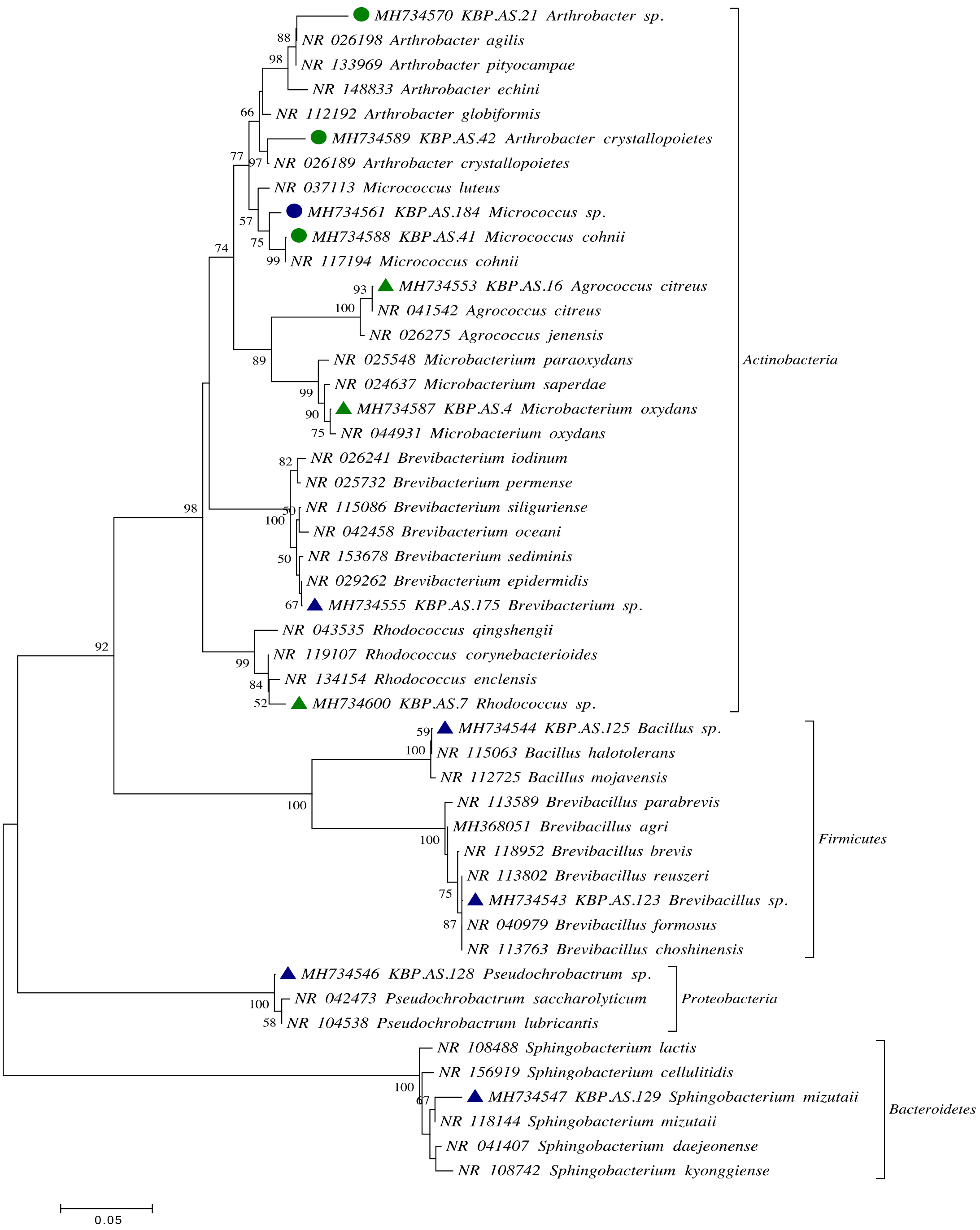

Figure S4. Phylogenetic analysis of 16S rRNA genes of the strains isolated from desert soils which were sequenced using 537r primer. The tree was constructed using the Neighbor-Joining method with the use of the most closely related sequences from GenBank. The number of taxa—45. The number of significant characters—342. The number of bootstrap replicates—500. Confidence values of branching are given for cases of 50% and higher. Circles indicate strains isolated from the Sahara desert, triangles—the Gibson desert; green and blue colours indicate strains isolated at 25 and 10 ℃, respectively. The scale under the tree indicates the number of phylogenetically significant changes of nucleotides.

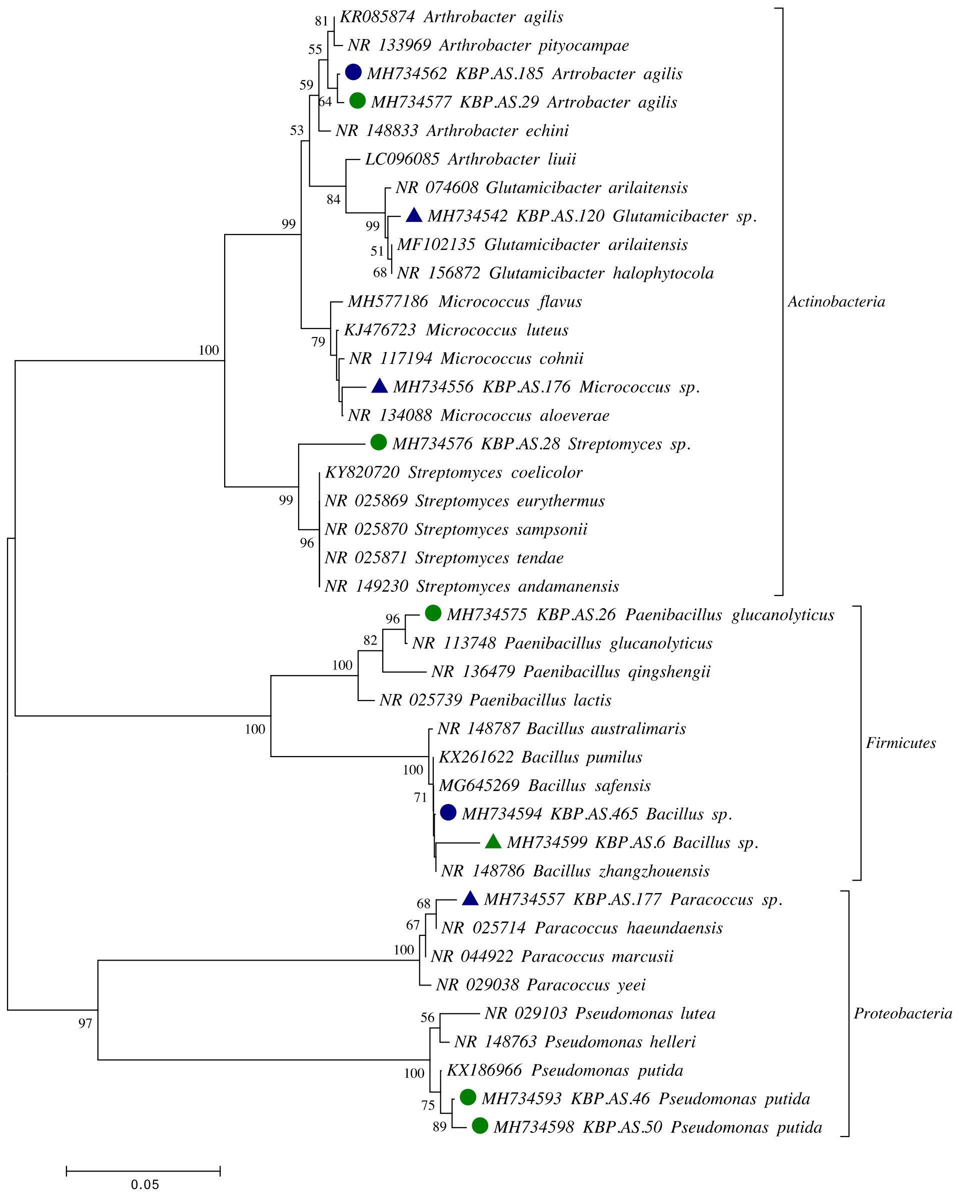

Figure S5. Phylogenetic analisis of 16S rRNA genes of the strains isolated from desert soils which were sequenced using 805r primer. The tree was constructed using the Neighbor-Joining method with the use of the most closely related sequences from GenBank. The number of taxa—40. The number of significant characters—382. The number of bootstrap replicates—500. Confidence vaules of branching are given for cases of 50% and higher. Circles indicate strains isolated from the Sahara desert, triangles—the Gibson desert; green and blue colours indicate strains isolated at 25 and 10 ℃, respectively. The scale under the tree indicates the number of phylogenetical significant changes of nucleotides.

AIMS Press

© 2018 the Author(s), licensee AIMS Press. This is an open access article distributed under the terms of the Creative Commons Attribution License (http://creativecommons.org/licenses/by/4.0)
